# Experimental study of fluid-thermal-structural interactions in a Mach-10 compression corner using super-ellipse-based photogrammetry

**DOI:** 10.1007/s00348-026-04209-y

**Published:** 2026-04-22

**Authors:** Travis A. Duchene, Antonio G. Schöneich, Brett F. Bathel, Joshua M. Weisberger, Gregory M. Buck, Daniel J. Bodony, Stuart J. Laurence

**Affiliations:** 1 Department of Aerospace Engineering, University of Maryland, College Park, MD 20742 USA; 2 Advanced Measurements & Data Systems Branch, NASA Langley Research Center, Hampton, VA 23681 USA; 3 Department of Aerospace Engineering, University of Illinois Urbana-Champaign, Urbana, IL 61801 USA

## Abstract

An experimental study is conducted of the fluid-thermal-structural interaction of a clamped compliant panel exposed to the intense shock-wave/boundary-layer interaction (SWBLI) induced by a compression ramp at Mach 10. Initial measurements of the underlying flowfield with a rigid ramp showed the incoming boundary layer to be transitional, and the SWBLI was observed to vary from attached to fully separated as the ramp angle was increased from 10$^\circ$ to 30$^\circ$. For the compliant panel, a sealed cavity behind the panel allowed the effects of pressure-differential induced strains to be studied in the context of characterizing surface response to the aero-thermal load. Full-field, time-resolved panel deformations were measured using high-speed photogrammetry enabled by a new high-fidelity marker-tracking routine, which was shown to outperform existing methods. Substantial static panel deformations (of the order of several times the panel thickness) were produced by the intense aero-thermal loading environment. These deformations, combined with induced thermal and pressure gradients across the panel, were found to significantly modify the nature of existing panel modes (both the frequency and the displacement distributions) and introduce new, irregular mode shapes not predicted by classical clamped-plate theory; SolidWorks^®^ simulations were performed to demonstrate that these new mode shapes were a result of the underlying panel curvature. Increasing the ramp angle resulted in a wider variety of panel modes becoming excited, while increasing the pressure differential across the panel typically produced further increases in modal frequencies and decreases in vibrational amplitudes. The transient panel response was characterized and it was found that the lower frequency mode shapes tended to gradually increase in vibrational frequency as the panel heated up and further deformed; however, higher frequency modes ($f \gtrsim 3 \hspace{.075 cm} \textrm{kHz}$) generally showed the opposite behavior. Furthermore, as the panel deformed through the test time, the average vibrational spectra root-mean-square power was generally found to monotonically decrease.

## Introduction

A present challenge in the development of reusable hypersonic vehicles is the ability to accurately characterize the intense aero-thermal-mechanical loading experienced at high speeds. Specifically, the design requirements for such vehicles usually favor long, slender configurations and necessitate lightweight and durable structural components (Mcnamara and Friedmann 2011). Shortcomings in predicting the aerothermal environment and the resulting structural responses of the coupled fluid-thermal-structural interaction (FTSI) have resulted in the over-design of current thermal protection systems and structural design margins (Eason and Spottswood 2013). Particularly, assessing the effects of localized heating and pressure loading caused by shock-wave/boundary-layer interactions (SWBLIs) on vehicle panels remains paramount as these can lead to boundary-layer separation with a corresponding low-frequency unsteady motion capable of coupling with compliant surface structures (Zuchowski 2010, 2012; Spottswood et al. 2013; Clemens and Narayanaswamy 2014).

Earlier developments in the modeling of high-speed fluid-structural interactions (FSIs) and FTSIs made use of simple aerodynamic models suitable for single panels (Dowell 1966; Gee and Sipic 1999; Librescu et al. 2004; LaFontaine et al. 2016), wings (Budiansky and Mayers 1956; Gupta et al. 2017), and conceptual vehicles (Heeg et al. 1993a, b). One of the major predictions in the FSI context was that a pinned panel experiences limit cycle oscillations rather than structural failure at hypersonic pressure loading conditions (Dowell 1966). More recent FSI work has shown that structural vibrations are able to couple with and alter the downstream flowfield, upstream unsteady shock dynamics, and flow separation behavior (Clemens and Narayanaswamy 2014; Pham et al. 2018; Eitner et al. 2022; Ahn et al. 2025). However, when including thermal effects, this coupling behavior becomes much more complicated; Budiansky and Mayers (1956) noted that the heating of wing elements significantly reduces the torsional stiffness, leading to a reduction in the vibrational frequency of surface elements. Further, both LaFontaine et al. (2016) and Riley and McNamara (2017) studied the two-way aerothermoelastic coupling and found an underlying importance of nonlinear temperature-dependent structural response. Few experiments, though, have examined FSI and FTSI at hypersonic conditions, particularly under SWBLI excitation. Among the critical remaining needs for an accurate characterization of SWBLI-induced FTSI is validation-quality experimental data, including measurements of the relevant fluid features and flexible panel response.

The two-dimensional compression-ramp-induced SWBLI is a canonical geometry that is well described at low supersonic Mach numbers (Dolling 2001; Ganapathisubramani et al. 2007; Gaitonde 2015); however, the same configuration has received less attention for hypersonic flight conditions, at which the effects of heating and low-frequency flow unsteadiness are expected to be amplified. From an FTSI perspective, Whalen et al. (2019) found severe pressure fluctuations near the shear-layer reattachment point for separated SWBLIs at Mach 6 on this geometry; with a compliant panel, there were static deformations on the order of panel thickness and vibrational frequency shifting, likely caused by the thermal stresses introduced by the temperature differential between the compliant panel and the support (Whalen et al. 2020). In measurements on a similar configuration with a variable back pressure, Schöneich et al. (2023) found that an increased pressure differential across the compliant panel introduced additional stresses and reduced the vibrational response, resulting in a reduction of pressure fluctuations upstream of the corner for nominally transitional incoming boundary layers. A downshifting in structural natural frequencies was attributed to increased temperature differentials, but the unavailability of flow visualization meant that it was not possible to assess how the potential shifting of the flow re-attachment point on the compliant panel contributed to changes in the mechanical response.

Various experimental methods exist for characterizing the mechanical response of flexible surfaces on a test geometry. Casper et al. (2019) and Pandey et al. (2023) used accelerometers to measure the vibrational response of a compliant panel to boundary-layer transition on a 7$^\circ$ half-cone at $M_{\infty } =5, 6,$ and $8$; however, accelerometer-based methods are limited in spatial resolution and can introduce changes in the structural natural frequencies of the panel due to their non-negligible mass. Single-point capacitive probe measurements, like those employed by Tripathi et al. (2021), are non-intrusive but similarly limited in achievable spatial resolution. If the compliant structure is optically accessible, global image-based techniques such as photogrammetry or Digital Image Correlation (DIC) can be highly desirable: these are non-intrusive and provide full-field measurements at the high frame rates enabled by modern high-speed cameras. In photogrammetry, surface motions are measured via a 3D reconstruction of specific tracked features among multiple camera views, whereas, in DIC, full-field surface displacements and strains are computed by correlating textured patterns between image frames (typically 2D or stereo). Both DIC (Banks et al. 2015; Beberniss and Ehrhardt 2017; Ahn et al. 2022) and photogrammetry (Whalen et al. 2020; Schöneich et al. 2023) have seen use in high-speed FTSI experiments, each having its own advantages and disadvantages. DIC approaches, while highly accurate, are computationally expensive and, being reference-image-based methods, do not allow the absolute locations of markers to be determined. Photogrammetry techniques can also be made highly accurate and are able to provide absolute marker locations, enabling measurements of both higher-frequency surface vibrations and long-term quasi-static deformations; however, existing methods in the literature have proved to be inadequate to accurately measure some of these higher-frequency and small amplitude vibrations.

Thus, in this work, we employ experiments to gage the response of a fully-clamped thin compliant panel in a compression ramp-induced Mach-10 SWBLI, i.e., the same configuration studied at Mach 6 by Whalen et al. (2020) and Schöneich et al. (2023). This higher Mach number condition is of interest as the stronger shocks can be expected to produce especially intense unsteady pressure loadings and heating. High-speed, high-resolution photogrammetry of the compliant panel is used to measure its shape and frequency response, and focusing schlieren is used to measure off-body flow structures. This article is organized as follows. In Sect. 2, we detail the test facility, model, and diagnostic techniques for the experiments performed. Section 3 describes and characterizes a newly developed high-fidelity marker-tracking photogrammetry routine for measuring the response of the compliant panel. In Sect. 4, we investigate the characteristics of the SWBLI on the baseline rigid ramp and present findings on the nonlinear structural response of the compliant panel to the SWBLI. Finally, Sect. 5 summarizes the main conclusions of this work.

## Experimental setup

### Facility

The experiments were conducted in the 31-Inch Mach-10 Air Tunnel of the Langley Aerothermodynamic Laboratory. This is a conventional blow-down wind tunnel that uses heated dry air as the test gas. Steady flow times of up to two minutes are realizable, though typical run times in the present experiments were limited to approximately 4 s. The reservoir conditions for the present study gave stagnation temperatures of $972.5 \pm 2.7$ K and stagnation pressures of $93.4 \pm 0.2$ bar, corresponding to a unit Reynolds number of $6.44 \pm 0.04 \times 10^6 \mathrm {m^{-1}}$ (see Table 1 for typical freestream test conditions). Freestream conditions were computed using the method of Hollis (1996). The tunnel has a closed 31 $\times$ 31 in. test section with a water-cooled, contoured nozzle to provide freestream Mach numbers ranging from 9.6 to 10. The exit flow is quite uniform with uncertainty in the freestream conditions typically around $\pm 1\%$ (Miller 1992). A side-loading, hydraulically powered injection mechanism is used to place the test article into the flow, and can vary the angle of attack from $-90^\circ$ to $90^\circ$, though the present experiments were all performed at zero angle of attack. The test section has optical access through the top, bottom, and side windows, allowing for simultaneous focusing schlieren and photogrammetry measurements. Further details of this facility can be found in Micol (1995).

**Table 1:** Run conditions

Parameter	Value
$M_\infty$	$9.95$
$Re_\infty$, m$^{-1}$	$6.44\times 10^{6}$
$p_\infty$, kPa	$2.28\times 10^{-1}$
$\rho _\infty$, kg/m$^{3}$	$1.62\times 10^{-2}$
$T_\infty$, K	49.1
$v_\infty$, m/s	1394.1

Before a given run, the upstream section of the facility is filled to a pressure of 100 psia and preheated to a stagnation temperature of 950 K. The hot air is then blown through the nozzle and test section for a short time in order to promote thermal uniformity of the test flow, resulting in a small degree of pre-heating of the test section. Next, the test section, nozzle, and settling chamber are pulled down to low-pressure conditions by opening a valve to an adjoining vacuum sphere. The model is subsequently injected into the test section to collect flow-off data. The model is then retracted into the injector box, at which point a valve upstream of the settling chamber starts the tunnel. Once the desired freestream conditions are reached, the model is injected into the test section centerline, signaling $t_0$ of the test time.

### Model

**Fig. 1:**
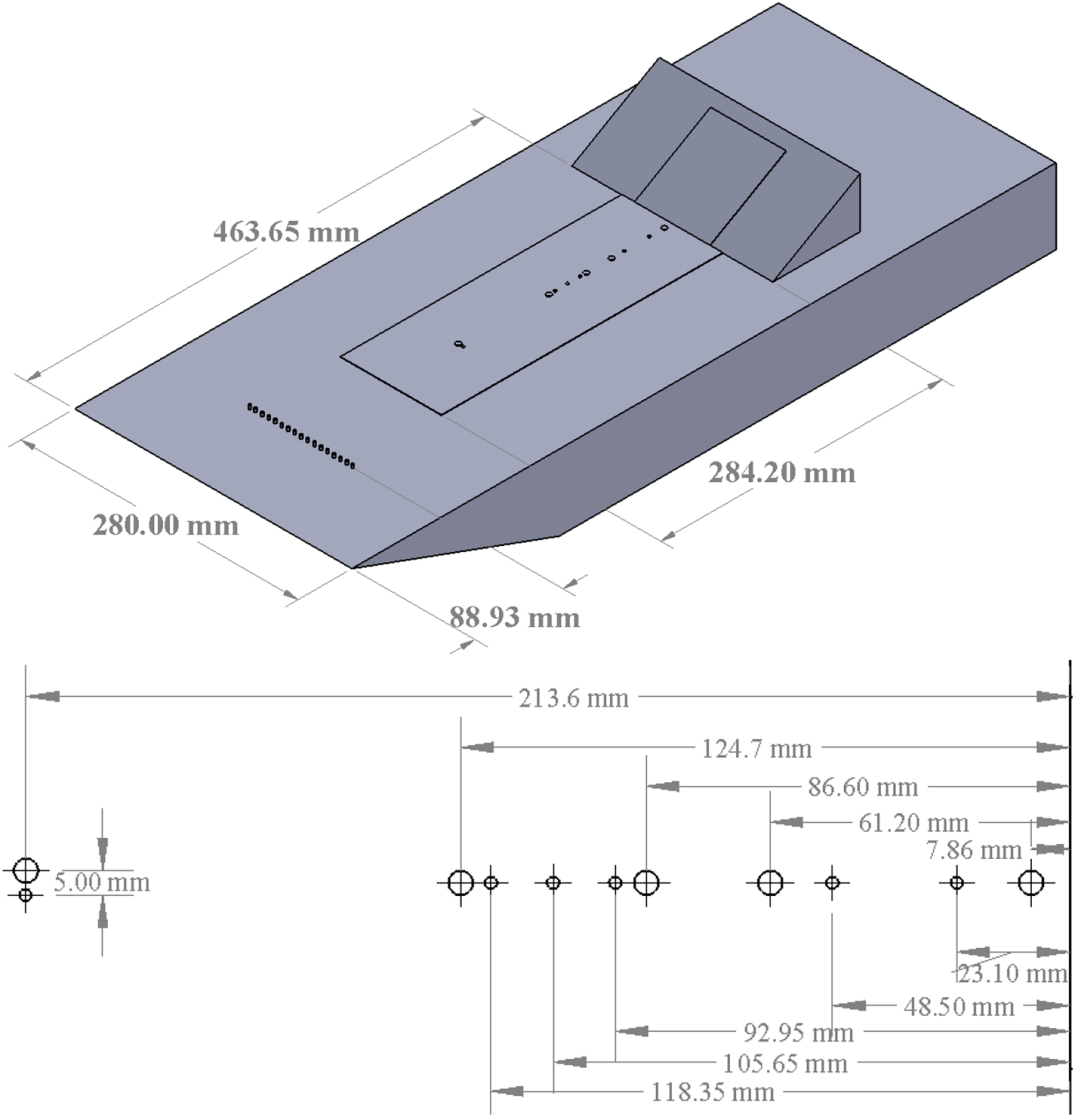
(Top) CAD rendering of flat-plate/ramp model and (bottom) layout of pressure sensors (PCBs^®^ are large markers, Kulites^®^ are small markers) along the model centerline. The vertical line on the right indicates the ramp corner

**Fig. 2:**
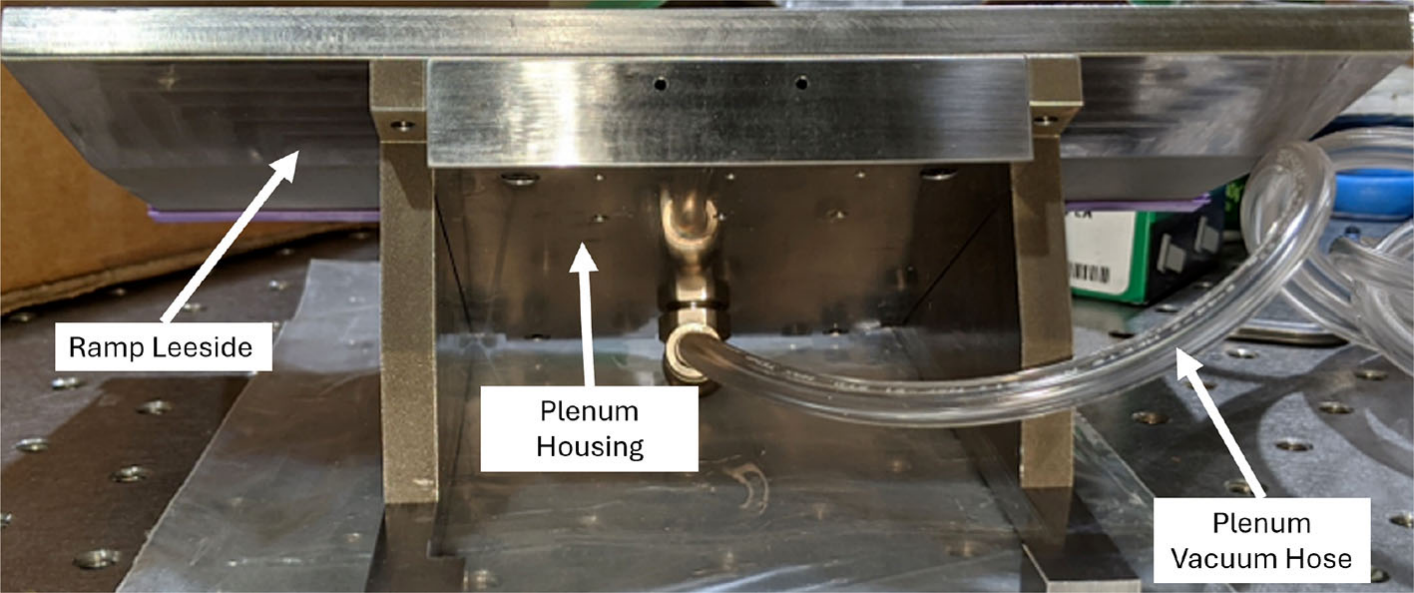
Rear view of ramp with plenum box housing and vacuum port

The test article was a flat-plate/ramp model, intended to produce a nominally two-dimensional SWBLI; a CAD rendering of the model is shown in the top image of Fig. 1. The base of the model consisted of a 280 mm wide by 711 mm long flat-plate with a sharp leading edge and a 101.6-mm-wide by 284.2-mm-long channel along the centerline for exchangeable inserts. In the present experiments, an insert with Kulite^®^ and PCB^®^ pressure transducers was used with its leading edge positioned 179.5 mm downstream of the model leading edge. A 15–5 PH/H1025 (AMS 5659) stainless steel ramp was placed 463.7 mm downstream of the flat-plate leading edge to generate a compression corner. This ramp measured 203 mm wide by 102 mm long with a rectangular cavity measuring 76.2 mm wide by 88.9 mm long machined out of the back, resulting in a 0.5 mm thick, fully clamped compliant panel embedded in the surface of the ramp. A separate 17–4 PH stainless steel rigid ramp was also fabricated that measured 177.8 mm wide by 101.6 mm long and could be mounted at the same streamwise location as the compliant ramp model. Both compliant and rigid ramps were tested, the latter to determine the baseline flow state. Although the rigid ramp was slightly narrower than the compliant one, we nevertheless expect the flow along the centerline (where the rigid-ramp measurements were made) to be unaffected and remain nominally two-dimensional. The outer sections of the ramp were designed such that, when the ramp assembly was inserted into the flat plate, the leading edge of the ramp was flush with the surface of the plate. The upstream edge of the compliant panel was co-located with the corner of the compression ramp (with a sharp trailing edge on the upstream insert to enable this). The ramps were placed on interchangeable angled brackets to produce nominal ramp angles of $10^\circ$, $20^\circ$, and $30^\circ$, spanning flow conditions from attached to fully separated SWBLIs.

A sealed plenum box was used to create a pressure-controlled environment on the back-side of the panel, allowing the effects of a pressure differential on the structural dynamics to be assessed (a photograph of this setup is shown in Fig. 2). The cavity generated by the plenum box measured 15.7 mm deep and was controlled by a 15 psia (103 kPa) pressure calibration unit. For each ramp angle, the back pressure was varied between a vacuum and the inviscid static, post-oblique shock pressures, $p_2$, calculated as 1.6, 5.0, and 10.2 kPa for the $10^\circ$, $20^\circ$, and $30^\circ$ ramps, respectively. Table 2 gives the pressure differential values ($\Delta p = p_2 - p_{\textrm{plenum}}$) tested for each ramp angle (a positive pressure differential indicates a higher pressure on the wind-side of the panel), giving a total of 12 combinations. Although, in reality, the pressure on the ramp will be nonuniform, the quoted $\Delta p$ values nevertheless provide a consistent characterization of the relative degree of purely mechanical strain present across the panel.

**Table 2:** Panel pressure differentials for each compression ramp angle

Ramp angle	$\Delta p$ [kPa]
$10^\circ$	$0$, $0.5$, $1.1$, $1.4$
$20^\circ$	$0$, $1.7$, $3.4$, $4.8$
$30^\circ$	$0$, $3.4$, $6.8$, $9.9$

A spanwise array of 17 boundary-layer trips were placed 88.9 mm downstream of the leading edge to promote the development of a turbulent boundary-layer near the ramp region. Each tripping element had a square diamond shape with a side length of 2.16 mm and a height of 3.05 mm; the center-to-center spacing was 6.6 mm apart with the corner of the diamond oriented into the flow.

The flat plate was instrumented with six flush-mounted piezoresistive Kulite^®^ pressure transducers (useful for low-frequency measurements where $f \lesssim 25$ kHz) sampled at $200$ kHz, and five piezoelectric PCB^®^ 132-B38 pressure transducers (suitable for high-frequency measurements where $f \gtrsim 15$ kHz), sampled at $2$ MHz; the locations of these sensors are shown in the lower part of Fig. 1. Note that the streamwise coordinate, $x$, is referenced to the corner, and we will use negative values hereinafter to indicate the position along the flat plate upstream of the corner. The plenum box was instrumented with two XCE-062 Kulite^®^ pressure transducers to measure the mean pressure and static pressure fluctuations inside the cavity behind the panel. Data sampling was triggered such that the PCBs^®^ and Kulites^®^ began sampling 0.2 s after the model reached the test section centerline.

**Table 3:** Nominal wind-off panel modes, $\Delta p = 0$

Mode shape	$f_{theory}$ [kHz]	$f_{vibrometer}$ [kHz]
(1,1)	$$0.67$$	$$0.51 $$
(2,1)	$$1.19$$	$$1.09 $$
(1,2)	$$1.43$$	$$1.16 $$
(2,2)	$$1.94$$	$$1.75 $$
(3,1)	$$1.96$$	$$1.99 $$
(1,3)	$$2.07$$	$$2.35 $$

Pre-campaign modal testing of the compliant panel was conducted using a Polytec PSV-300 scanning vibrometer. Panel natural frequencies were characterized with a sampling frequency of 100 kHz. The backside of the panel was enclosed with the sealed plenum box to create a pressure controlled environment. Four interrogation locations were chosen that lay away from nodes of the six lowest-frequency modes. For zero pressure gradient across the panel, Table 3 shows the measured natural frequencies of these six modes together with corresponding theoretical results from Freydin and Dowell (2021). The theoretical predictions are generally higher than the measured values. This is thought to be a result of non-ideal structural boundary conditions, in which panel edges are not perfectly clamped but are more akin to a clamped-pinned boundary condition. Further, Schöneich et al. (2021) noted that the natural frequencies increased with an increasing pressure differential. The increased pressure differentials were also found to decrease the likelihood of thermal buckling caused by elevated panel temperatures.

### Focusing schlieren

**Fig. 3:**
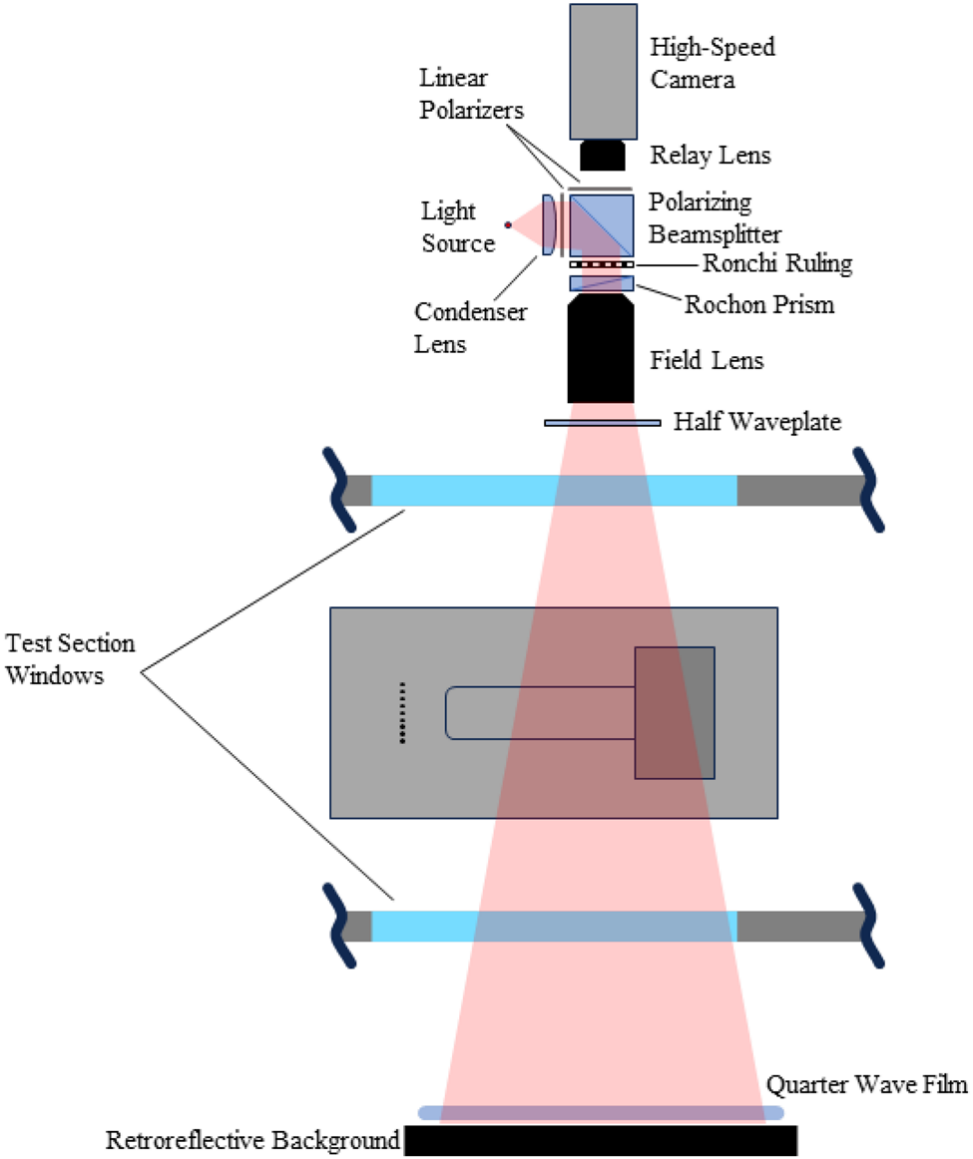
Schematic of self-aligned focusing schlieren (SAFS) setup

The path-integrated nature of conventional schlieren systems is not suitable for isolating unsteady features near the centerline of the model in this, nominally two-dimensional SWBLI. Instead, a self-aligned focusing schlieren (SAFS) system was used to confine the schlieren response to a narrow depth-of-field, effectively reducing the influence of density gradients outside the plane of interest (Bathel and Weisberger 2021, 2022; Bathel et al. 2024). In the present implementation (shown in Fig. 3), a laser light source (Cavitar Cavilux HF) was loosely collimated and diffused through a condenser lens with a diffusing surface, before passing through a linear polarizer and reflecting onto the main system optical axis by a polarizing beamsplitter cube. The linear vertically polarized light back-illuminated a Ronchi ruling (Data Optics) that was then projected onto a retroreflective background (3M Scotchlite 7610) on the opposite side of the tunnel by a field lens (Nikon, 300 mm focal length). Between the Ronchi ruling and field lens, the light passed through a polarizing Rochon prism (United Crystals, glass/quartz, 7.5 arcmin splitting) where the linear vertically polarized light remained unrefracted. Just prior to the retroreflective background, a thin piece of quarter-wave film was oriented such that it converted the linear vertically polarized light to right-circularly polarized light. Upon retro-reflection, the handedness of the light was converted to left-circularly polarized, and upon return passage through the quarter-wave film, to linear horizontally polarized light. The field lens then formed an image of the returning light back onto the Ronchi ruling, where the incoming light was refracted slightly by the Rochon prism because of its horizontal polarization state, with translation of the prism along the optical axis providing an adjustment of the sensitivity of the system akin to the knife-edge cutoff in a conventional schlieren system. The light then passed through the polarizing beamsplitter, through another linear polarizer to increase image contrast, and an image of the density object was formed at the camera sensor plane (Photron SA-Z) through the use of a relay lens (Niko AF Nikkor, 50 mm, 1:1.4D). To provide sensitivity to both the horizontal and vertical density gradients in the flow simultaneously, the Ronchi ruling and Rochon prism were rotated to $45^\circ$ relative to the freestream flow direction. Various Ronchi ruling line spacings were used throughout testing depending on the required sensitivity. Images were recorded at 75,000 fps with a resolution of 256 $\times$ 1024 pixels, effective exposure times of 250 ns, and an image magnification of 6.5 pixels/mm. Note in Fig. 3 that the half-wave plate was positioned between the field lens and top window of the test section. Thermal stress imparted to the test section windows during facility preheating resulted in modification of the polarization state of light passing through the top and bottom windows, reducing signal-to-noise. By adjusting the orientation of this half-wave plate and the quarter-wave film below the test section, the signal-to-noise of the schlieren images could be adjusted and optimized.

### Photogrammetry

**Fig. 4:**
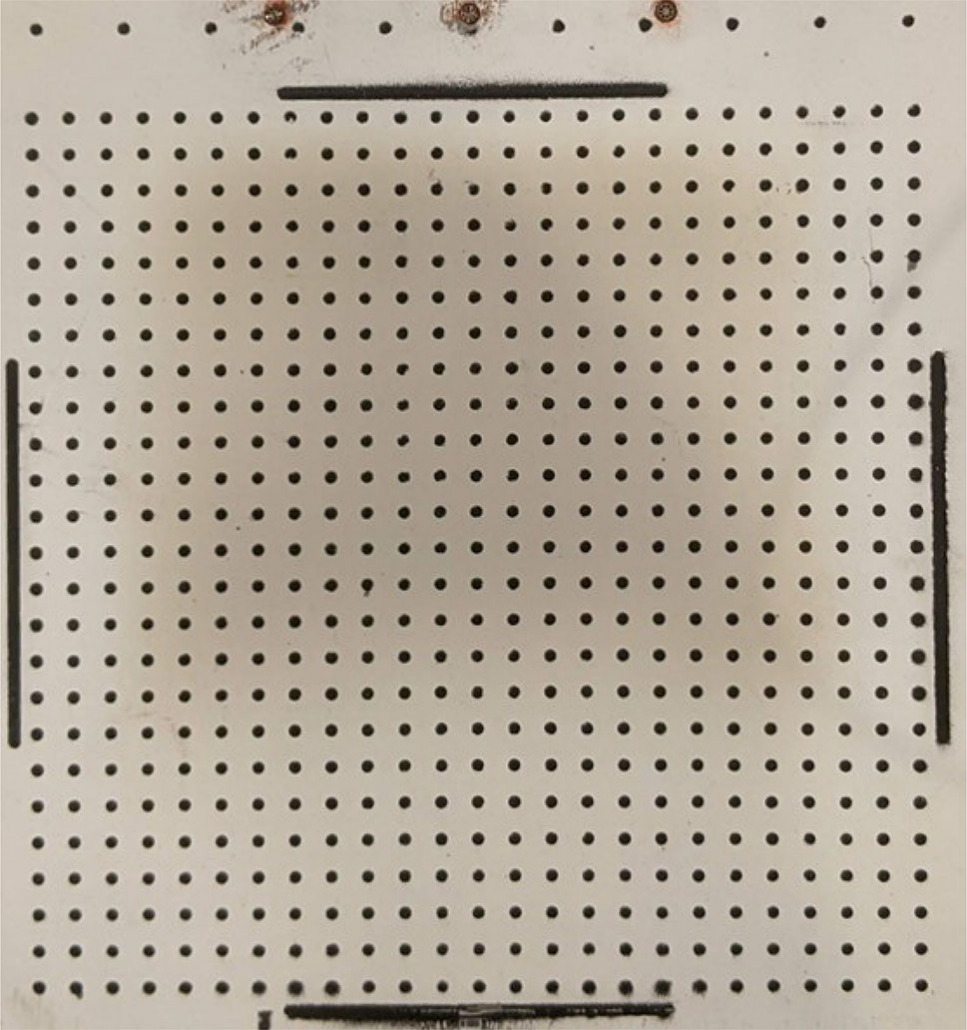
Ramp with marker grid painted on the compliant region

Measurements of the out-of-plane panel motion were performed using marker-based photogrammetry. Originally developed as an optical technique for topographical measurements, photogrammetry has received considerable attention as a measurement technique in aerospace applications: Baqersad et al. (2017) provides a substantial review on the usefulness of photogrammetry to inform structural response in such applications. For hypersonic FTSI’s especially, many geometries of interest involve three-dimensional structural motion due to fluid-structural coupling. Thus, two-dimensional surface deformation measurements, like those achievable through schlieren imaging (Baqersad et al. 2017; Currao et al. 2019), are no longer suitable, motivating the present approach.

A few experimental considerations are necessary for successful photogrammetry measurements. Marker-tracking photogrammetry begins with the precise application of markers to the compliant panel; these markers are usually applied as painted dots, as shown in Fig. 4 (Whalen et al. 2020; Schöneich et al. 2023). In our setup, a grid of markers was painted on the exposed surface of the compliant panel. The elastic modulus of the paint layer is orders of magnitude smaller than that of AMS 5659 steel, so the paint layer is expected to have a negligible impact on the panel response. To ensure circularity of the markers, we employed a stencil methodology similar to that of Whalen et al. (2020). One 25 $\times$ 25 grid of 1.27 mm diameter circular markers was painted onto the panel, as shown in Fig. 4. The top row of 10 markers was painted onto the rigid portion of the ramp to act as reference markers for tracking the bulk model motion. Two or more cameras are needed in order to construct three-dimensional marker motion from two-dimensional images. The conversion of two separate sets of two-dimensional locations in image space into a singular set of three-dimensional locations in physical space is then typically done through calibration, such as the bundle-adjustment algorithm (Walker et al. 2009). For accurate determination of the marker centroid, Whalen et al. (2020) found that it is preferable to have at least 6 camera pixels span the marker diameter, though as we will see, this requirement can be relaxed with the method proposed here.

Four 460 nm Luminus PT-120 big-chip light emitting diodes (LED) were each passed through a condenser lens, reflected off a parabolic reflector, and passed through a diffuser to illuminate the panel with focused, uniform light. Two Photron SA-Z high-speed cameras visualized the illuminated panel with an angular separation of $74.4^\circ$. Each high-speed camera was equipped with an Edmund Optics # 89–772 band-pass filter that transmitted the LED light while blocking light from the SAFS system. Each filter was mounted to a Nikon 105 mm f/2 lens, providing a resolution of 768 x 768 pixels. Images were recorded at 30 kfps using an exposure time between $10-20\,\mathrm \mu s$; due to camera memory constraints, only approximately 1 s of run-time data were collected. The average time between model injection and the beginning of image recording was typically around 0.5 s. Calibration of the camera apparatus was performed for each ramp angle by imaging a LaVision QR3-169−6.4 calibration target mounted to the surface of the ramp. The model was injected fully to the test section centerline, and then retracted to 30 discrete locations (average displacement of $\sim 1.15$ mm), at which images were taken by each camera. The bundle-adjustment algorithm provided by Walker et al. (2009) was then used to constrain the camera parameters. Wind-off images of the painted panel were captured at vacuum conditions (with zero pressure differential across the panel) before each run to enable monitoring of any plastic deformation occurring during the test campaign. To estimate the systemic errors that may arise from the calibration, residuals were calculated to a planar fit to the marker locations on the calibration target at a given position. The standard deviation of these residuals was $0.3\,\mathrm \mu m$ with a maximum absolute value of $0.6\,\mathrm \mu m$.

**Fig. 5:**
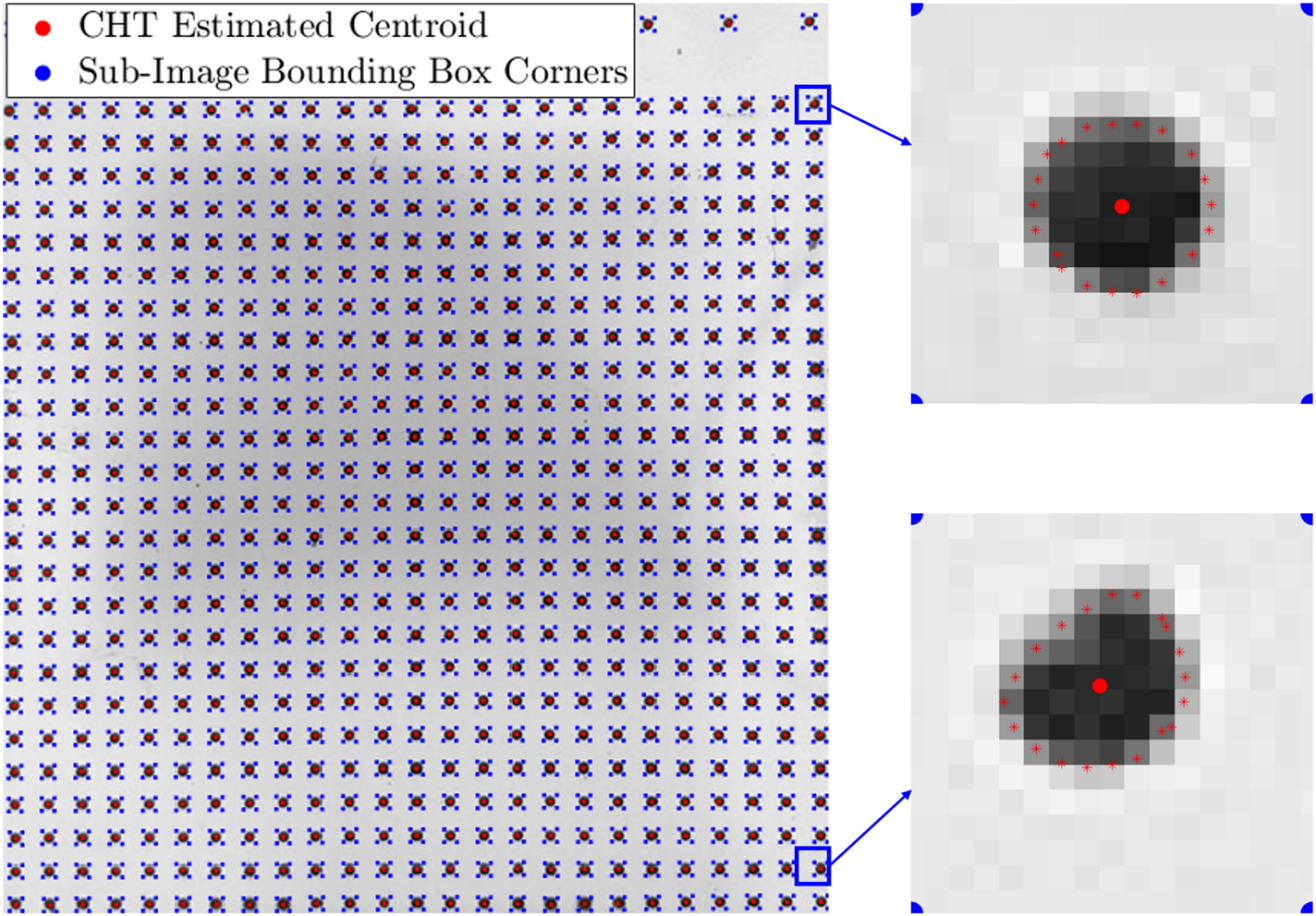
(Left) CHT identified centroids with the corresponding bounding box corners, and (right) example sub-images generated with the corresponding subpixel edge-point cloud

## Super-ellipse marker-tracking approach

### Preliminary considerations

In the previous studies of Whalen et al. (2020) and Schöneich et al. (2023), out-of-plane panel displacements were found to be on the order of tens of microns, with even smaller deformations for lower ramp angles. These minute displacements motivate the need for highly-accurate marker-tracking algorithms to resolve the out-of-plane structural motion. A computationally inexpensive technique for measuring the marker centroid location in a given image is a center-of-mass localization technique. This technique, however, is not capable of providing the high levels of accuracy needed to measure small panel deformations. To improve on this method, Whalen et al. (2020) developed a high-accuracy model-fitting routine to identify the centers of blurred, pixelated elliptical markers, using a $tanh$ representation of the intensity profile. This technique was shown to be able to resolve marker center locations to within $\sim 0.012$ pixels for marker diameters greater than 6 pixels; however, as it relies on the Nelder-Mead algorithm for a nonlinear least-squares fit to the marker, it is computationally expensive. Furthermore, the accuracy decreases for markers smaller than 6 pixels in diameter.

To provide an improved balance between computational efficiency and accuracy, here we introduce a hybrid methodology that employs a highly efficient but lower-fidelity initial localization (ellipse tracking) to provide an initial guess for a higher-accuracy iterative localization (super-ellipse fitting). These techniques are adaptations of optical-tracking methods recently introduced for free-flight aerodynamic measurements (Duchene and Laurence 2025).

### Sub-image identification and subpixel edge detection

The first step of the present algorithm is to separate a given image (such as shown in Fig. 4) into $X$ sub-images, where $X$ is the number of individual markers. A circular Hough Transform (CHT) is used to provide initial estimates of all marker centroid locations in the original image, which provides the basis for subsequent sub-division into sub-images via bounding boxes. The Hough Transform is an ideal first-guess-type approach as it is robust in the presence of noise, occlusion, and varying nonuniform image illumination. Similarly, this algorithm is flexible in identifying multiple circles of varying radii in a single image.

In each sub-image, a subpixel edge detection is performed to identify the outline of the marker, similar in principle to the edge-tracking method developed by Laurence (2012) for aerodynamic measurements of free-flying objects. Here, a modified version of the partial-area subpixel method developed by Trujillo-Pino et al. (2013) is used. This method was tested and validated for edges that can be readily approximated as second-order curves. The subpixel routine is performed on each sub-image, resulting in a locus of edge points as shown in Fig. 5. For ideal grids with no artifacts or background noise, the only identified points would lie on the edge of the given marker. In practice, however, image artifacts could result in erroneous detections. The Density-Based Spatial Clustering of Applications with Noise (DBSCAN) algorithm of Ester et al. (1996) was thus implemented to remove such outliers. This is a shape flexible algorithm, meaning it is suitable for datasets in which the grid markers, and their corresponding point-clouds, appear elliptical rather than perfectly circular. Similarly, this algorithm does not require a predefined number of clusters in which to search, which is necessary for other clustering algorithms like the K-means method (Kanungo et al. 2002).

**Fig. 6:**
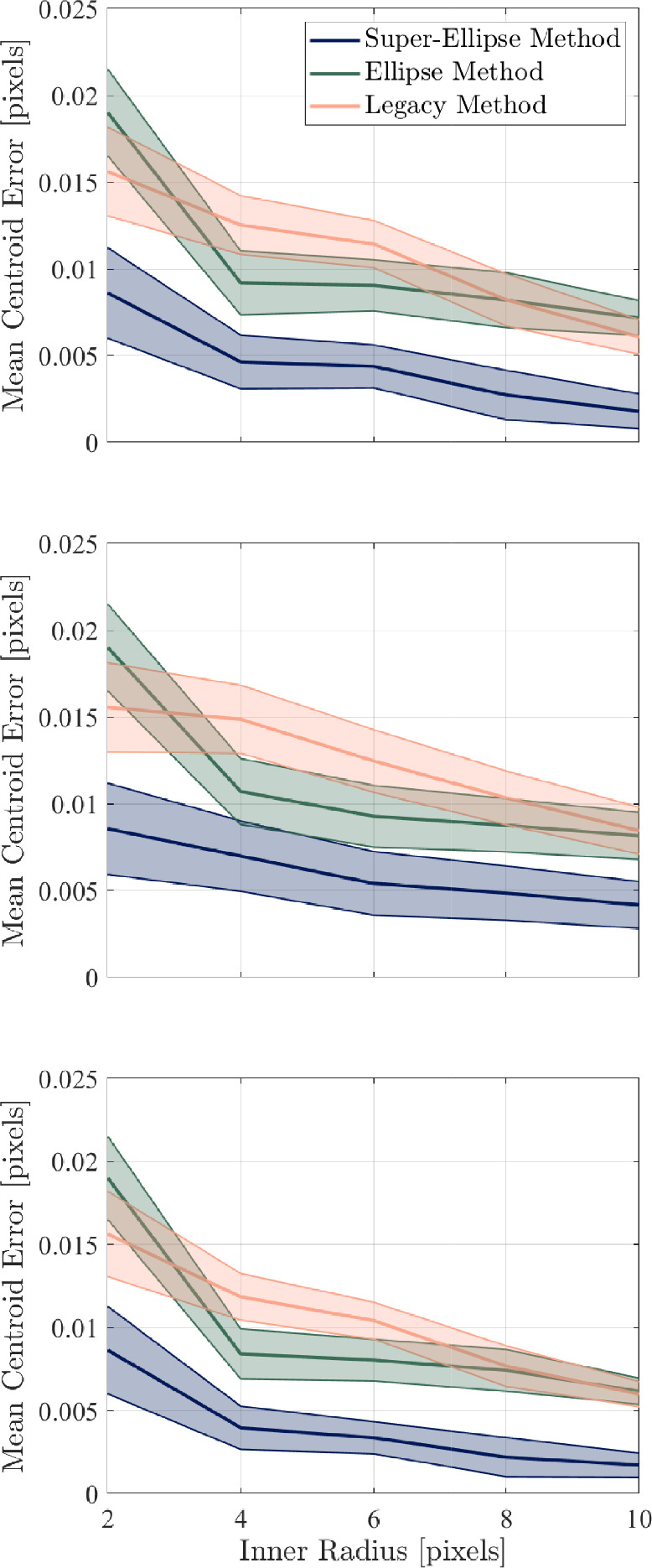
Results of artificial marker analysis, showing the marker-tracking algorithm performance (mean RMS error with $2\sigma$ bounds) as a function of marker radii for (top) circular markers for $\sigma _n = 2\%$, (middle) eccentric ($e = 0.75$) markers for $\sigma _n = 2\%$, and (bottom) circular markers with $\sigma _n = 0.1\%$

### Ellipse and super-ellipse marker-tracking algorithms

With the edge points ($x_e, y_e$) identified and outliers removed, we can proceed to the fitting step. The lower-fidelity ellipse method fits an equation of the form

1 $$\begin{aligned} &  \frac{1}{a}((x_e-x_0)\cos \phi +(y_e-y_0)\sin \phi )^{2} +\nonumber \\ &  \qquad \frac{1}{b}((y_e-y_0)\cos \phi -(x_e-x_0)\sin \phi )^{2}=1, \end{aligned}$$

to the edge points, where $x_0$, $y_0$, $\phi$, $a$, and $b$ are the unknowns. The numerically stable direct least-squares fit from Halir and Flusser (1998) is employed to determine these parameters given the locus of edge points. The values $x_0$ and $y_0$ are the main parameters of interest, as they represent the centroid of the marker, while the orientation of the fitted ellipse is determined through the $\phi$ parameter.

The higher-fidelity super-ellipse tracking technique solves the equation

2 $$\begin{aligned} &  \frac{1}{a}((x_e-x_0)\cos \phi +(y_e-y_0)\sin \phi )^{2/n} +\nonumber \\ &  \qquad \frac{1}{b}((y_e-y_0)\cos \phi -(x_e-x_0)\sin \phi )^{2/n}=1, \end{aligned}$$

where the $n$ parameter now allows the fitted shape to conform better to non-strictly elliptical shapes. The method works by ingesting the edge points and the output of the ellipse-method parameters ($a$, $b$, $\phi$, $x_0$, and $y_0$) as an initial guess; the initial value of $n$ is set to unity. The goodness of fit is then evaluated at the N edge points using the average centroid-to-edge orthogonal distance as

3 $$\begin{aligned} err = \frac{1}{N}\sum _{i=1}^{N}|r_i - s_i|. \end{aligned}$$

Here, $r_i$ is the radial distance from the origin of the edge points aligned to the super-ellipse coordinates and $s_i$ is a point sampled on the super-ellipse taken at the same internal angle as $r_i$. Using the built-in MATLAB nonlinear least-squares optimization function, the trust-region reflective algorithm is used to iterate to the best-fit super-ellipse parameters that minimize the error metric.

### Characterization with artificially generated markers

To assess the accuracy of the tracking algorithms, characterization using artificially generated marker grids was performed. The validation test involved creating 100 10x10 grids with markers of varying sizes, eccentricities ($e$) and image noise ($\sigma _n$) - with each marker being randomly displaced between successive images by up to 3 pixels - to give 30 different test conditions. The marker sizes assessed had radii of 2, 4, 6, 8, and 10 pixels; the eccentricities tested were 0 (i.e., a circle) and 0.75. Varying levels of additive Gaussian noise were also included ($\sigma _n =$ 0.1%, 1%, and 2%) to assess the robustness of the tracking algorithm. The grids consisted of 8-bit images with a black (zero intensity) background and white (255 intensity) markers, each described as an ellipse with a centroid $x_0,y_0$ and semi-major and semi-minor axes $a$ and $b$, respectively. Gaussian smoothing was applied to the image before noise addition to simulate more realistic experimental images (i.e., blurring). Every marker in each grid was tracked using the legacy algorithm of Whalen et al. (2020), the ellipse algorithm, and super-ellipse algorithm, and the root-mean-square (RMS) error in the center location was recorded.

Beginning first with the circular ($e = 0$) markers, the upper-part of Fig. 6 shows how each algorithm performs as a function of $r_{marker}$ in the highest noise ($\sigma _n = 2\%$) environment. The error bars represent the 95% confidence interval, over all markers. As we might expect, the accuracy increases with increasing marker size, for each method. For the legacy algorithm, the RMS error decreases from $\sim$0.015 pixels for $r_{marker} = 2$ pixels to $\sim$0.006 pixels for $r_{marker} = 10$ pixels. The ellipse method shows similar overall error levels, but the performance of the super-ellipse method is significantly better, with the mean RMS error decreasing from $\sim$0.009 to $\sim$0.002 pixels as the marker radius is increased from 2 to 10 pixels. Similar trends are seen for the $e = 0.75$ case shown in the middle part of Fig. 6: introducing nonzero eccentricity slightly increases the error bounds, but otherwise has little effect on accuracy for all of these techniques. The legacy and ellipse-tracking methods exhibit similar accuracy, with the latter method seeming to be the slightly better choice except for the smallest marker size. For both marker types, decreasing the image noise predominantly decreased the uncertainty bound, but had little effect on the mean error. This is demonstrated in the lower part of Fig. 6, where corresponding results for circular markers with $\sigma _n = 0.1\%$ are shown. In terms of computational efficiency, processing 100 images on a single-core workstation with the legacy method took approximately 40 min, whereas the corresponding computational times for the ellipse and super-ellipse methods were 16 min and 28 min.

**Fig. 7:**
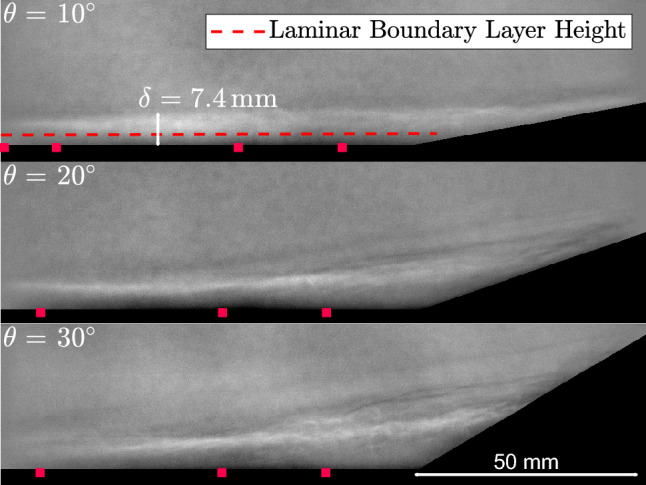
Sample background subtracted instantaneous focusing schlieren images for each ramp angle: (top) $\theta = 10^\circ$, (middle) $\theta = 20^\circ$, and (bottom) $\theta = 30^\circ$ all at $\Delta p = 0$. Red boxes indicate the location of Kulite^®^ sensors

## Results

### Underlying SWBLI flowfield

To better contextualize the later FTSI results, we first aim to characterize the underlying SWBLI flowfield, including the incoming boundary-layer state. For this we rely primarily on measurements taken on the rigid-ramp configuration, the exception being for the schlieren visualizations: these were not analyzed for the rigid ramp since the interrogation region was upstream of the compression corner. The basic flow structures in the schlieren are not expected to vary significantly between rigid and compliant configurations, however. Sample background subtracted schlieren images for the three ramp angles are shown in Fig. 7, with red points indicating the location of Kulite^®^ sensors. For the 10$^\circ$ ramp, transitional structures were clearly visible in the upstream boundary layer, and the edge of the boundary layer (identified as $\delta = 7.4 \hspace{.05cm} \textrm{mm}$ from the rapid change in image intensity) clearly extends above the theoretical laminar boundary-layer height indicated; these structures did not appear to be fully turbulent, however. For the 10$^\circ$ ramp, there was no clear evidence of flow separation, though it is possible that a region of incipient separation is present at the corner. For the 20$^\circ$ ramp, a limited separation region appears that becomes more fully developed for the 30$^\circ$ ramp. In both cases, unsteady transitional structures were observed along the separated shear layer.

**Fig. 8:**
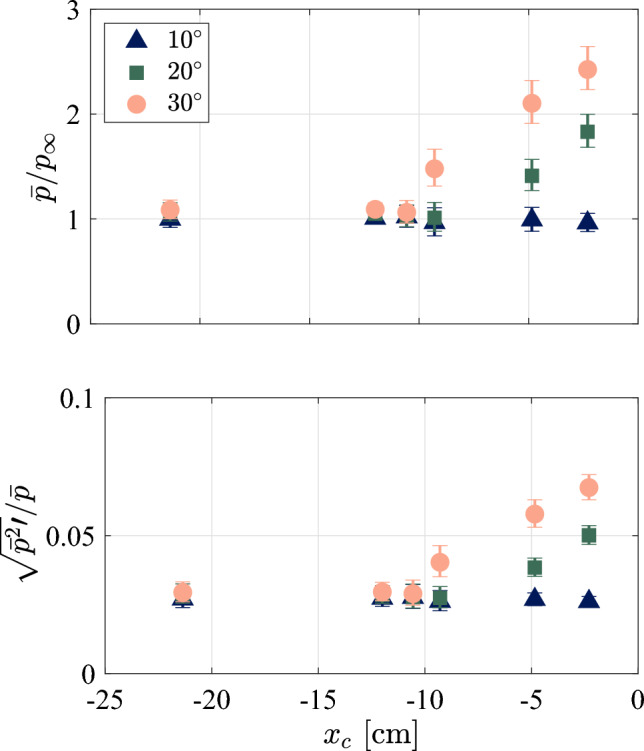
(Top) Mean pressure measurements normalized by the free stream pressure and (bottom) RMS pressure measurements on the rigid ramp normalized by the mean pressure for each rigid ramp angle, $\theta$

The spatial distribution of the mean and temporally varying wall pressure upstream of the corner gives further insight into the nature of the hypersonic SWBLI. The normalized mean pressures and RMS fluctuation values over the full test time from the centerline Kulite^®^ sensors are presented for the rigid ramps in Fig. 8, with error bars corresponding to the 95% confidence interval in the mean. Only frequencies less than 40 kHz were used in the evaluation of the RMS fluctuation values shown. For the mean pressure, the 10$^\circ$ ramp values remain essentially constant, confirming that the flow remains attached until the most downstream sensor ($x_c=-2.3\,\textrm{cm}$). For the other two ramp angles, we see a significant rise in the pressures as the flow approaches the compression corner. As we would expect, the higher ramp angle produces a larger pressure rise and a more extended region of upstream influence: the increase in $\bar{p}/p_{\infty }$ between $x_c = -10.5 \textrm{cm}$ and $x_c = -9.3 \textrm{cm}$ indicates that the influence of the separation region extends to roughly $10\,\mathrm cm$ upstream of the ramp corner. We thus estimate the separation length, $L_{\textrm{sep}}$, in this case as 10 cm, and expect this value to be accurate to within 7%. For the 20$^\circ$ ramp, the sparsity of pressure measurements between $x_c = -9.3\,\mathrm cm$ and $-4.8\,\mathrm cm$ renders it difficult to make a precise estimate of $L_{\textrm{sep}}$. Linearly interpolating the $\overline{p}/p_\infty$ values from the last two sensors to unity gives $L_{\textrm{sep}}$ = 7.6 cm. Alternatively, we can examine the schlieren images and identify the location at which the $\theta = 20^\circ$ boundary layer begins to diverge from the $\theta = 10^\circ$ one; this latter method gives a separation length of 7.4 cm. We thus assign a value of $L_{\textrm{sep}}$ = 7.4 cm in this case and expect that this value is accurate to within 10%. The mean-scaled RMS pressure variations show similar trends to the mean pressures. In the upstream boundary layer, the variation is approximately 3% of the mean value, which remains roughly constant until the corner for the 10$^\circ$ ramp. For the other compression angles, the rise in this quantity upstream of the corner indicates that the pressure variations grow more rapidly than the mean pressure beneath the separated flow region.

**Fig. 9:**
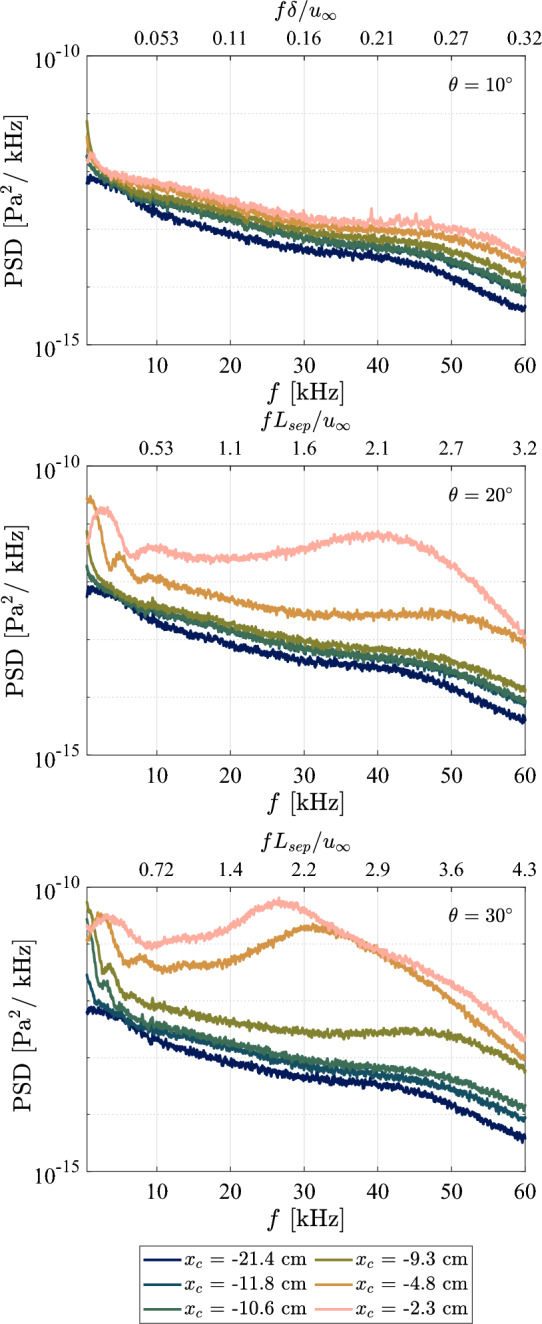
Upstream pressure spectra at each Kulite^®^ sensor location for the (top) $10^\circ$, (middle) $20^\circ$, and (bottom) $30^\circ$ rigid ramps

**Fig. 10:**
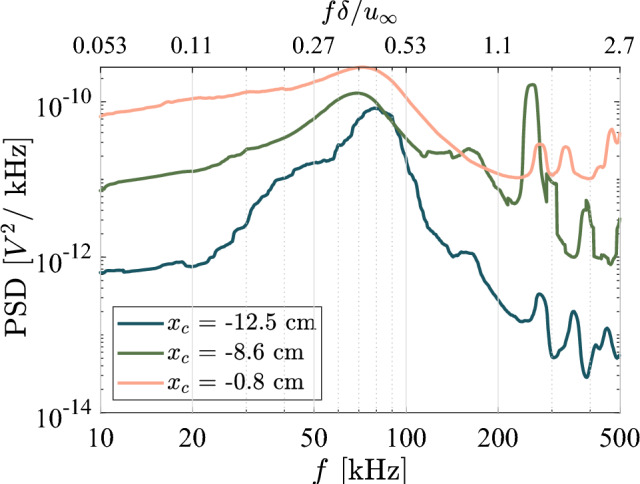
Power spectral densities of pressure fluctuation measurements from PCB^®^ sensors along the upstream flat plate for $\theta = 10^\circ$

To determine the contributions to these pressure variations in frequency space, in Fig. 9 we show spectra from the Kulite^®^ measurements for each ramp angle. Here, the power spectral density (PSD) was calculated using Welch’s method, averaging 10,000-point segments of the pressure signals with 50% overlap. These spectra are presented in terms of both dimensional frequency and nondimensional Strouhal number. The relevant length scale chosen for calculating the Strouhal number is the upstream boundary-layer thickness for the 10$^\circ$ ramp and $L_\textrm{sep}$ for the 20$^\circ$ and 30$^\circ$ ramps. For the 10$^\circ$ ramp, a general growth across all frequencies is observed moving downstream. There are no obvious spectral peaks, though a slight elevation near $50\,\mathrm kHz$ is visible. The second mode frequency at these conditions (estimated as $u_e/2\delta$) would be approximately $220\,\mathrm kHz$, so these disturbances may correspond to the first mode. It is to be noted, however, that Kulite^®^ sensors are affected by an acoustic resonance that renders measurements above $\sim 25\,\mathrm kHz$ quantitatively unreliable (though qualitative trends are still broadly valid). To gain a clearer picture of these upstream disturbances, in Fig. 10 we show high-frequency PCB spectra at select locations. Now a clear peak near 70–$80\,\mathrm kHz$ emerges: this peak grows modestly moving downstream, but we observe much more substantial growth in the background broadband levels, indicating that the boundary layer is on the verge of transitioning to turbulence at the most downstream station.

In the Kulite^®^ spectra for the 20$^\circ$ and $30^\circ$ ramps in Fig. 9, we see broad peaks develop within the separated region that both grow in amplitude and reduce in frequency moving downstream ($50\,\mathrm kHz$ dropping respectively to $40\,\mathrm kHz$ for 20$^\circ$ and $\sim 30\,\mathrm kHz$ for 30$^\circ$). These peaks may be associated with shear layer disturbances similar to those seen, for example, on a cone/flare geometry by Butler and Laurence (2022), reinforcing that the flow has not yet transitioned to a fully turbulent state. Some low-frequency peaks ($f\lesssim 5\,\mathrm kHz$) are also visible in the spectra of the transducers within the separated regions. These may correspond to “breathing” of the separation bubble, and are notable in that they lie at sufficiently low frequencies to potentially couple with the natural modes of the downstream panel. Similar peaks near $f L_\textrm{sep} / u_\infty \lesssim 0.3$ have been associated with low-frequency separation shock motions in earlier investigations (Dussauge et al. 2006; Priebe and Martín 2012; Whalen et al. 2019).

### Flow impingement location on compliant panel

**Fig. 11:**
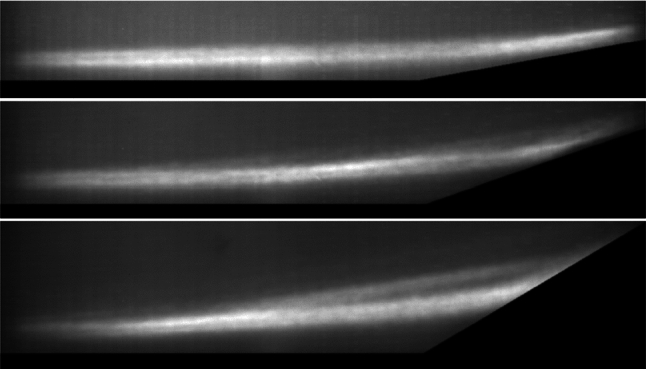
RMS image of 32 ms of run time for (top) $\theta = 10^{\circ }$, (middle) $\theta = 20^{\circ }$, and (bottom) $\theta = 30^{\circ }$, all at $\Delta p = 0$

In the previous study of Whalen et al. (2020), it was found that the shear-layer impingement location on a compliant region in SWBLI-induced FTSIs can play a dominant role in determining the degree of panel excitation. Focusing schlieren imagery can provide a qualitative understanding of the flow environment over the compliant panel region and, in particular, inform the impingement location of the most energetic upstream flow structures. In Fig. 11, we show derived schlieren images of the RMS intensity variation computed from a sequence of 2400 images for the three ramp angles. For the 10$^\circ$ ramp, the most energetic flow structures (visible as the bright band) lie close to the boundary-layer edge, while for the other two ramp angles it is displaced up onto the separated shear layer.

**Fig. 12:**
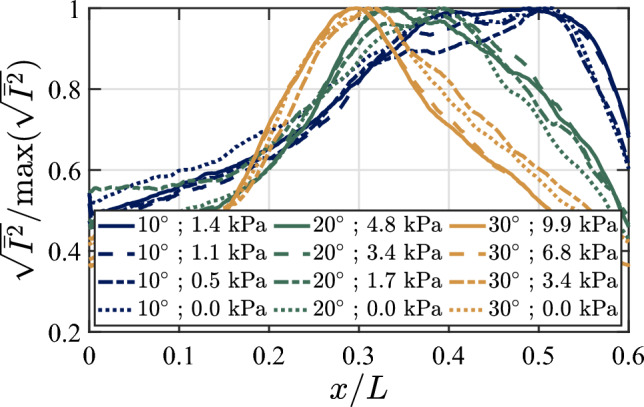
Normalized RMS of pixel intensity ($I$) as a function of the impingement location for the most energetic flow structures on the compliant panel region for each ramp angle and pressure differential

We estimate the mean impingement location of these structures on the ramp by computing the wall-normal-averaged grayscale RMS for the row of pixels parallel to the ramp in the near-wall region, normalized by the maximum value, with results shown in Fig. 12. Two seemingly important trends are apparent as the ramp angle increases: first, the nominal impingement location moves further upstream on the compliant region, and second, the spread over which this impingement location occurs narrows. Both these trends are caused by the angle of the separated shear layer (or attached boundary-layer edge) increasing more slowly than the ramp angle. As the lowest ramp angle places the impingement location nearest the center of the panel, we might infer that, all else being equal, lower-order mode shapes with anti-node locations near the center (such as the (1,1) mode) will be preferentially excited for this configuration; in contrast, for the highest ramp angle we might expect that more complex, higher-order streamwise mode shapes will dominate the panel motion.

### Mean panel response

#### Comparison of marker-tracking methods

**Fig. 13:**
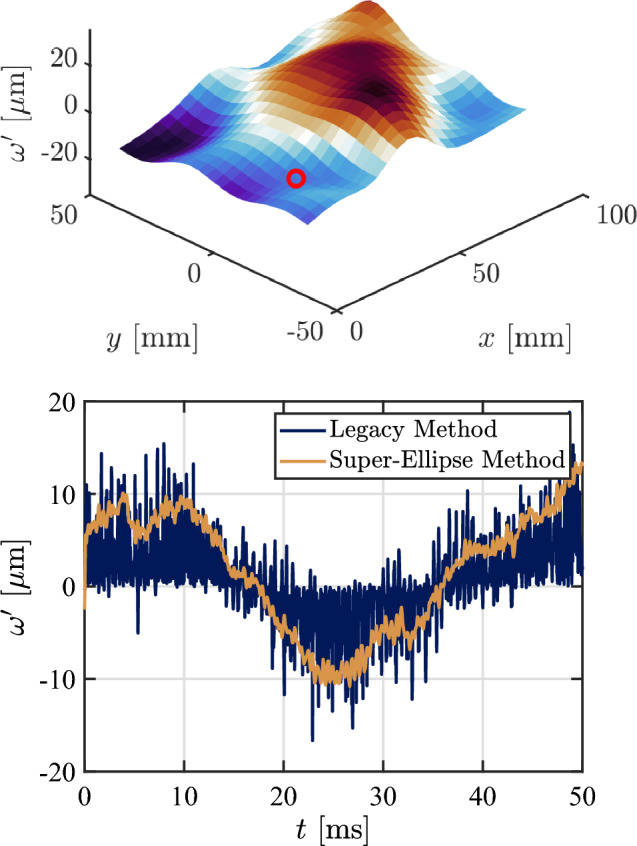
(Top) Instantaneous snapshot of mean-subtracted panel deformation, $\omega '$, and (bottom) time series of the highlighted data point using the legacy and newly developed marker-tracking methods

In order to demonstrate the usefulness of the super-ellipse technique in a more practical context, we first compare panel deformation measurements made with the new technique to those derived from the legacy method of Whalen et al. (2020). For both methods, we perform spatial Gaussian averaging (with $\sigma = 9.5$ mm) of the measured out-of-plane displacements, $\omega '$, in order to reduce measurement noise and account for variable paint thickness. The bulk panel motion is determined from reference markers and is subtracted out from the displacement data of all the compliant panel markers, allowing accurate measurements of the static and dynamic panel deformation. Figure 13 shows an instantaneous panel deformation map derived using the super-ellipse method for the $30^\circ$ ramp, together with representative time series from a single marker obtained with both techniques. We note that the newly developed method exhibits significantly reduced noise in the measurement of $\omega '(t)$ (on the order of $1\,\mathrm \mu m$), allowing the unsteady motion to be much more clearly discerned.

**Fig. 14:**
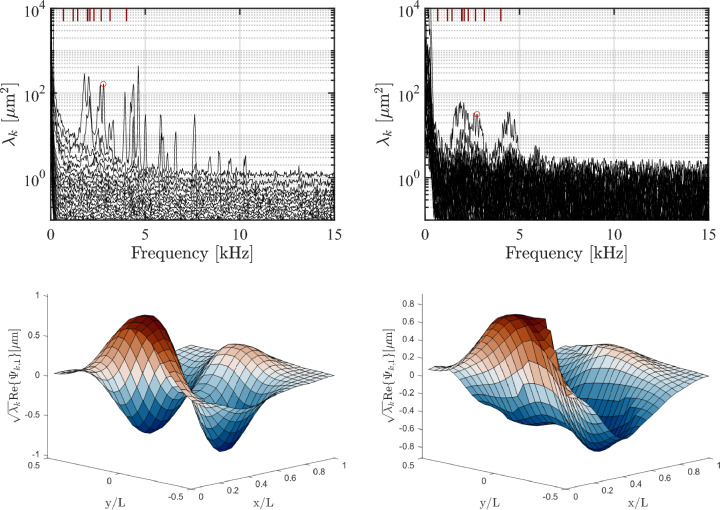
(Top) SPOD spectra for $\theta = 30^\circ , \Delta p= 0$ using (left) the super-ellipse marker-tracking algorithm and (right) the legacy marker-tracking algorithm; the respective mode shape for the indicated spectral peak is shown below each SPOD spectrum

To estimate the marker-tracking accuracy in the wind-tunnel environment more rigorously, we follow a procedure similar to Whalen et al. (2020). As no coherent panel motion for the $10^\circ$ ramp is detected above 6 kHz, we high-pass filter the time-series data of each marker and employ Parseval’s theorem together with the assumption that measurement error is a white noise process to obtain the expected measurement error. The standard deviations of the filtered data are multiplied by five to represent a high-confidence bound on the measurement error. The mean out-of-plane displacement error for the super-ellipse method is calculated as $\bar{\omega ^\prime } = 0.95\,\mathrm \mu m$ (0.006 pixels) compared to $\bar{\omega ^\prime } = 3.12\,\mathrm \mu m$ (0.019 pixels) for the legacy method.

The instantaneous deflection map in Fig. 13 shows some coherent features; however, there is no obviously dominating mode shape, suggesting that multiple modes are contributing to the deformation. In order to isolate these contributions in a mean sense, we make use of the spectral proper orthogonal decomposition (SPOD) algorithm reintroduced by Towne et al. (2018); this algorithm determines a set of orthogonal modes for each frequency based on an eigendecomposition of the cross-spectral density tensor formed by Fourier-transformed images. Compared to proper orthogonal decomposition (POD), which produces modes that are coherent only in space, SPOD is able to construct modes that are coherent in both space and time, thus representing dynamically significant features of the selected process. We take the out-of-plane displacements of grid markers as the input for the algorithm and obtain the frequencies and shapes of coherent patterns associated with the panel natural modes. The upper row of Fig. 14 show the frequency spectra obtained from the two tracking methods for the $30^\circ$ ramp with $\Delta p = 0$; the theoretical frequencies for the panel modes are indicated by the maroon ticks on the upper axis of each plot. In comparing the tracking methods, the super-ellipse method exhibits well-defined peaks with amplitudes on the order of $\lambda _k \simeq 10^2 \hspace{.05cm} \mathrm {\mu m^2}$ (where $\lambda _k$ represents the associated eigenvalue of the displacement signal), whereas the legacy method produces much more broadband peaks with low frequency resolution and reduced peak amplitudes. In the lower part of Fig. 14, we present the modal shape associated with the identified 2.78 kHz peak in each spectrum. The super-ellipse method gives a well-resolved spatial distribution of the mode shape, whereas the legacy approach has a noticeable amount of high-spatial-frequency noise which dilutes the precision in identifying the node and anti-node locations. This example is generally representative of the two methods and demonstrates the superior performance of the super-ellipse method in practical measurement environments. All subsequent results presented herein will thus use this method.

#### Mean static deformation and panel dynamics

**Fig. 15:**
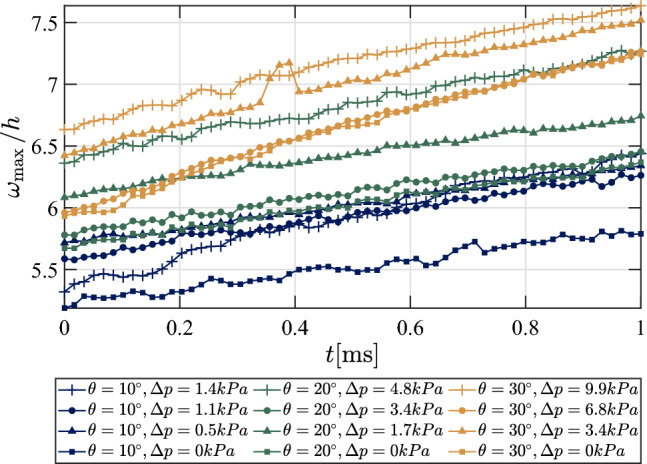
Temporal development of the maximum static deformation, $\omega ^\prime _{\textrm{max}}$, normalized by panel thickness, $h$, relative to the wind-off (zero-pressure-differential) shape for each ramp angle and pressure differential

Before investigating the compliant-panel dynamics any further, we first examine the temporal evolution of the “static” panel deformation for the various test conditions, as this will help inform interpretation of the dynamics. Relevant curves are shown in Fig. 15. This deflection was computed relative to the wind-off reference shape with no pressure differential across the panel (i.e., vacuum on both sides). The convention employed here is that a positive $\omega ^\prime$ corresponds to a displacement toward the lee side of the panel. In each case, we see a significant initial deflection ($\sim$ $6h = 3\,\mathrm mm$), followed by a further increase during the measurement time. The initial deflection is affected by both the pressure differential introduced following the reference measurement and the accumulated thermal stresses between the injection of the model and the beginning of the recording time. The trends for both these factors are generally as expected, with the initial deflection typically increasing with higher $\Delta p$ values and larger ramp angles (for which heating will be more intense). Considering the relatively limited variation with $\Delta p$ compared to the overall magnitude of $w'_{max}/h$, we can conclude that thermal stresses provide the dominant contribution to these static deflections. These deflections are substantially larger than those recorded at Mach 6 by Whalen et al. (2020), which were only up to 90% of the panel thickness. This difference can be attributed to the thinner panel and more intense heating environment in the present study.

To determine the likely temperature differential (between the compliant panel and supporting structure) responsible for these deflections, we performed finite-element simulations in SolidWorks^®^, in which the temperature differential $\delta T_\mathrm {panel-ramp}$ was varied between 0 K and 300 K for three choices of pressure differential between 0 kPa and 10 kPa; these are detailed in Appendix A. Based on these results, the magnitudes of the deflections in Fig. 15 are consistent with temperature differentials between 200 K and 300 K. Furthermore, these simulations confirm that the accumulated thermal stresses have a dominant effect over the pressure differential for the range of parameters considered here. Considering these large temperature differentials, we must also consider the possibility of thermal buckling having occurred at some point during the experiments (though it should also be noted that this would invalidate the assumptions in the simulations just described). In Schöneich et al. (2021), for example, theoretical calculations predicted that thermal buckling should occur for temperature differentials of approximately 30 K for $\Delta p$ = 0 kPa and 90 K for $\Delta p$ = 50 kPa under similar flow conditions. We thus examined both the time history of the displacement shape from the photogrammetry, looking for snap-through events, as well as the time-resolved spectra of the Kulite^®^ sensors in the plenum cavity, searching for significant drops in the (1,1) mode frequency, as evidence of buckling occurring. For all ramp types and $\Delta p$ conditions, no clear evidence of buckling was found which may provide further evidence that the effective panel boundary boundary conditions are diverging from fully clamped-clamped (as assumed in the calculations of Schöneich et al. (2021)). Nevertheless, given the likely temperature differentials involved, the possibility of thermal buckling having occurred at some point during these experiments cannot be fully ruled out.

The large panel deformations just noted can also be expected to have a significant influence on the panel mode shapes and frequencies (see, for example, Pinho et al. 2023). To gain a first-order estimate of these effects, we performed a SolidWorks^®^ frequency analysis of both a compliant panel with no deformation and deformed panels with various maximum deflections (5*h*, 6*h*, and 8*h*, corresponding the $2.5\,\mathrm mm$, $3.0\,\mathrm mm$, and $4.0\,\mathrm mm$). Further details are provided in Appendix B, together with the first five computed mode shapes and corresponding frequencies for each maximum deflection value. Note that this analysis does not account for the streamwise pressure and temperature gradients present in the experiments, so cannot be expected to reproduce the experimental results fully. Deforming the panel is seen to eliminate the (1,1) mode and increase the frequencies of the remaining conventional mode shapes, primarily the (1,2) and (2,1) modes. We also note the introduction of new, irregular mode shapes that are not present in the undeformed panel: a shape that resembles a rotated (2,2) mode (i.e., the 4th, 3rd, and 2nd computed mode shapes for the $2.5\,\mathrm mm$, $3.0\,\mathrm mm$, and $4.0\,\mathrm mm$ deformations, respectively), and two or three further unconventional mode shapes that exhibit a central antinode together with curved nodal lines (i.e., the 2nd and 5th modes for the $2.5\,\mathrm mm$ and $3.5\,\mathrm mm$ deformations, and the 4th mode for the $4.0\,\mathrm mm$ deformation).

**Fig. 16:**
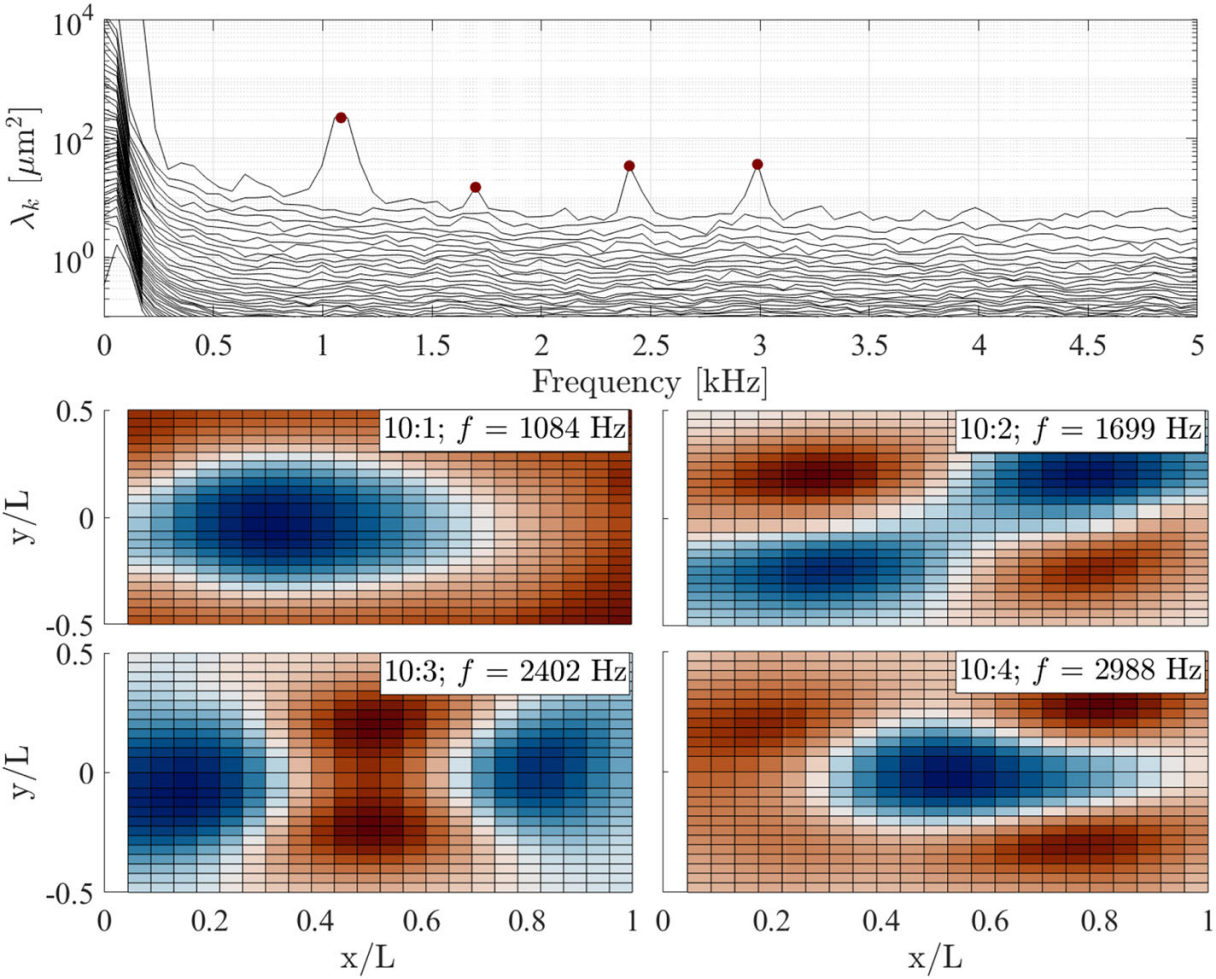
SPOD spectra and sample mode shapes in ascending order of $f$ for $\theta = 10^{\circ }$, $\Delta p = 0$ kPa

**Fig. 17:**
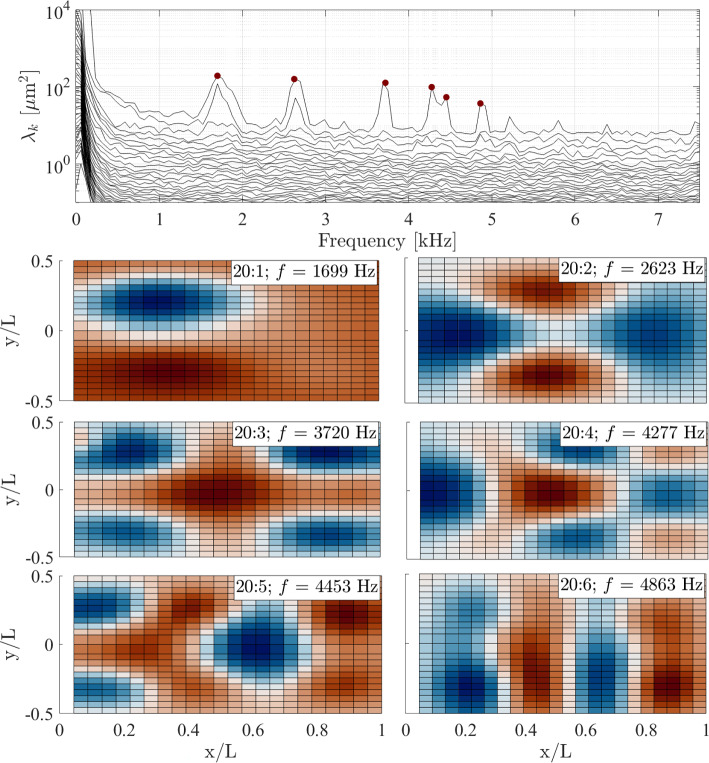
SPOD spectra and sample mode shapes in ascending order of $f$ for $\theta = 20^\circ$, $\Delta p = 0\text { kPa}$

**Fig. 18:**
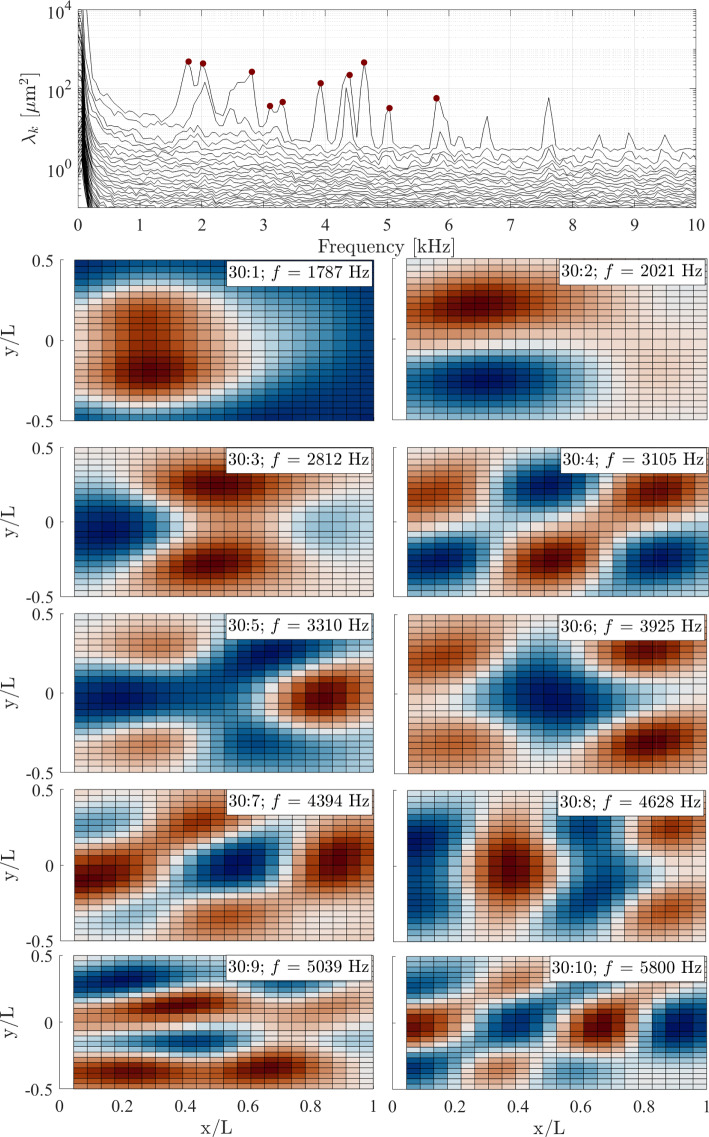
SPOD spectra and sample mode shapes in ascending order of $f$ for $\theta = 30^\circ$, $\Delta p = 0$ kPa

Now returning to the mode shapes determined experimentally via the SPOD analysis, these are presented in Figs. 16, 17, and 18, in each case with the respective SPOD spectrum, for ramp angles of 10$^\circ$, 20$^\circ$, and 30$^\circ$; all results presented are for zero $\Delta p$ (introducing a pressure difference changed the modal frequencies, as we will see shortly, but the mode shapes were generally unaffected). To begin, we note that in all cases (but particularly for the larger ramp angles) a significant number of excited modes below 10 kHz are present; this is relevant given that the low-frequency shock motion observed both here and in earlier works (Dussauge et al. 2006; Priebe and Martín 2012; Whalen et al. 2019) contains substantial energy within this frequency range. Because many of the derived mode shapes deviate from the conventional (m,n) natural modes for a fully clamped plate, we adopt a naming convention based on the frequency of the relevant peak in the SPOD spectrum for each ramp angle: for example, the 10:3 mode is that with the third-lowest-frequency peak in the SPOD spectrum for the 10$^\circ$ panel. In contrast to the computational predictions, the (1,1) mode does still seem to be present (10:1 and 30:1) but is highly distorted, with the antinode shifted substantially toward the upstream part of the panel; this can be assumed to result from the streamwise pressure and/or temperature gradients that the panel is subject to. This shifting upstream of the antinode may also be why the (1,1) mode is still substantially excited for the 30$^\circ$ ramp, despite Fig. 12 showing that the separated shear layer is impinging near $x/L=0.3$. A similar distortion can be seen in the (1,2) mode (labeled as 20:1 and 30:2). For both these distorted mode shapes, the frequencies increase significantly with increasing ramp angle (and also in comparison to the relevant wind-off frequencies obtained in the pre-tunnel tests, given in Table 3). Since this will also correspond to increasing static deflection, this general trend is consistent with the results of the simulations described in Appendix A. Some other conventional (m,n) mode shapes appear to be present, though not consistently across the various ramp angles, e.g., (2,2)$\rightarrow$10:2, (3,2)$\rightarrow$30:4, (2,3)$\rightarrow$30:5, (4,1)$\rightarrow$20:6 and 30:8, (3,3)$\rightarrow$20:4 and 30:7, (1,4)$\rightarrow$30:9, (4,3)$\rightarrow$30:10. The mid-frequency mode shapes, especially (2,2) and (3,2) (i.e., 10:2 and 30:4), tend to be less distorted than the (1,1) and (1,2) modes, though more significant distortions are again present in the higher-frequency modes: for example, the 20:6 and 30:8 shapes can only be tenuously identified as the (4,1) mode, and similarly for the 30:9 shape and the (1,4) mode.

In addition to these more familiar mode shapes, however, we also see some that deviate substantially from the expected regular (m,n) patterns. The ‘rotated (2,2)’ mode from the simulations is observed for all ramp angles (i.e., 10:3, 20:2, and 30:3) and at similar frequencies to the computational predictions. Another consistently present shape (10:4, 20:3, and 30:6) has antinodes at the panel center and distributed toward the four corners, and thus may correspond to one of the other irregular computed mode shapes (the frequencies would be most consistent with the 5th mode for the $3.0\,\mathrm mm$ and $4.0\,\mathrm mm$ deformations). In any case, substantial differences are observed in the panel dynamics here compared to the earlier Mach-6 measurements of Whalen et al. (2020), where the observed mode shapes followed the clamped-plate natural modes much more closely.

**Fig. 19:**
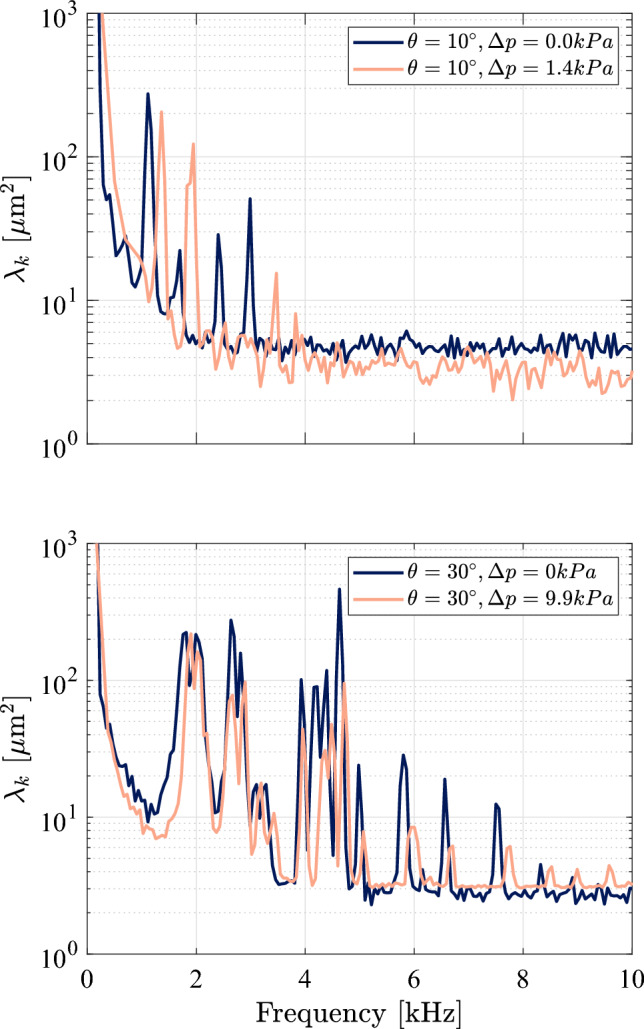
SPOD spectra for the (top) $\theta = 10^\circ$ ramp and (bottom) the $\theta = 30^\circ$ ramp angle at the lowest and highest $\Delta p$ values examined

Next, we employ the SPOD algorithm to compare the vibrational response at the highest and lowest pressure differentials for the same ramp angle (both the $10^\circ$ and $30^\circ$ ramps), as shown in Fig. 19. For the $10^\circ$ ramp in the upper graph, we see a noticeable increase in vibrational frequencies for the higher $\Delta p$ case as well as a reduction in the overall amplitude of $\lambda _k$, the one exception being the mode at $f \simeq 2$ kHz, which corresponds to the regular (2,2) mode shape. For the $30^\circ$ ramp, we see a similar reduction in the $\lambda _k$ peak values, most notably for the higher frequency mode shapes, with somewhat more modest increases in frequency than for the lower ramp angle. Generally, across all the ramp angles, the increase in frequency was most apparent for the lower-frequency mode shapes. Further, the higher $\Delta p$ case tended to dampen the overall amplitude of the higher-frequency modes more severely than the lower-frequency ones, suggesting that they are more strongly affected by the increased panel curvature. These results may also indicate that, for the weaker SWBLIs, the static pressure forcing plays a more significant role than the thermal loading in dictating structural response. Furthermore, in Fig. 15, the subsequent increase in deflection during the measurement time is indicative of additional thermal stresses introduced as the panel temperature continues to rise; although not uniformly the case, the curves for the higher $\Delta p$ cases do appear to have larger slopes, indicating that the maximum deflection is increasing more rapidly during the recorded time in these cases. This time-varying deflection motivates us to explore the time-frequency response of the structural motion in the following section.

### Temporal evolution of modal characteristics

**Fig. 20:**
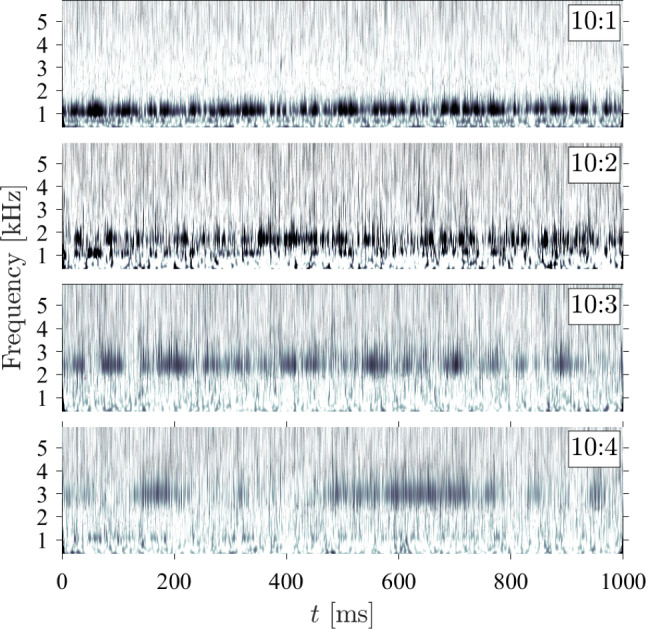
Wavelet-transformed modal projection coefficients of the first four modes for the $10^\circ$ ramp with $\Delta p = 0 \textrm{kPa}$

**Fig. 21:**
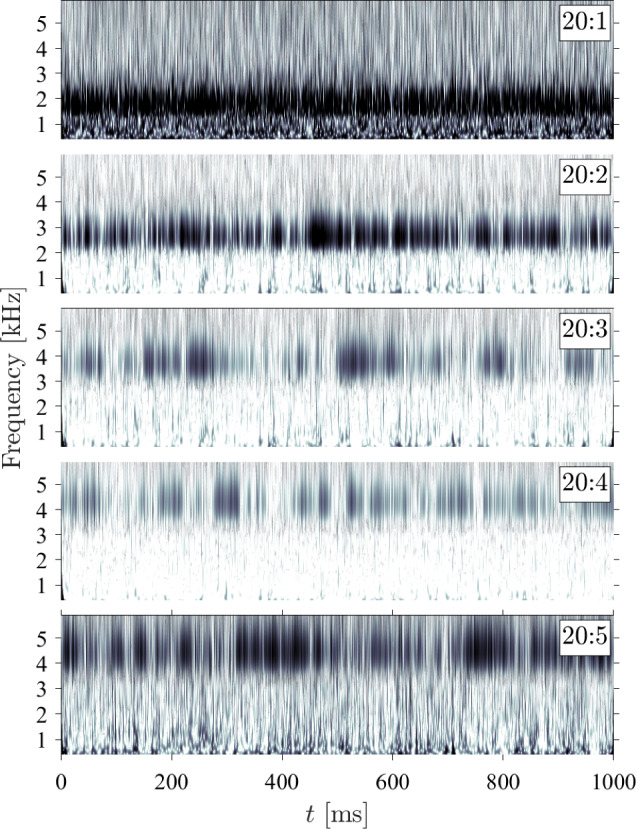
Wavelet-transformed modal projection coefficients of the first five modes for the $20^\circ$ ramp with $\Delta p = 0 \textrm{kPa}$

**Fig. 22:**
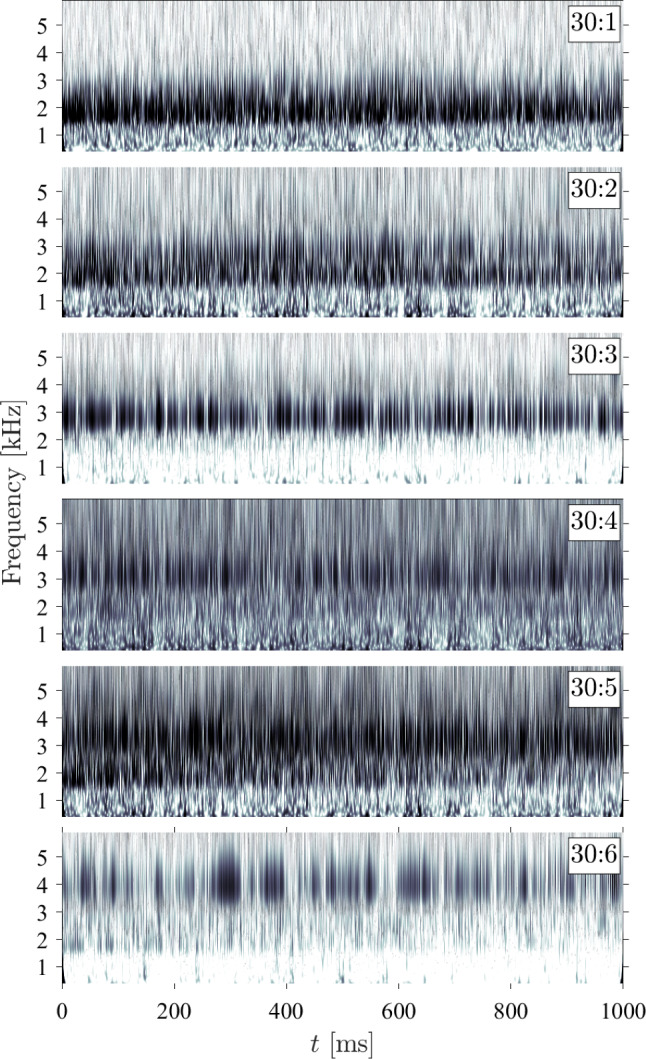
Wavelet-transformed modal projection coefficients of the first six modes for the $30^\circ$ ramp with $\Delta p = 0 \textrm{kPa}$

The SPOD analysis in Figs. 16 to 19 depicts the average panel dynamics over the recorded test time. As Fig. 15 makes clear, however, the base panel state is changing in time, which can also be expected to lead to temporal variations in the panel dynamics, in terms of both the vibratory frequency and power of each panel mode. To assess such variations, we first characterize the degree to which each mode is active throughout the recorded time. To do this, we employ a similar procedure to that of Young (1950), applying an inner-product projection of the instantaneous normalized panel state onto the excited mode shapes measured from the SPOD analysis. In Figs. 20, 21, and 22, we present the Morlet wavelet transforms of these projections for the first few excited modes (each on its own sub-plot) for each ramp angle with $\Delta p = 0$.

For the lowest ramp angle shown in Fig. 20, all modes show periods of activation (indicated by the darker regions) with varying intervals of quiescence in between, suggesting the panel is subject primarily to impulsive loading. The 10:1 mode is the most persistent, and exhibits a gradual upward shift in frequency to $f \simeq 1.2$ kHz; the relatively poor frequency resolution of this method (i.e., the inability to distinguish between closely spaced frequencies in the signal) prompts the use of a short time SPOD analysis, as shown later, for more conclusive evidence of frequency shifting throughout the recorded test time. Both the 10:2 and 10:3 modes (i.e., the standard and ’rotated’ (2,2) mode shapes, respectively) are fairly active, though they appear mostly as bursts of excitation rather than a continuous presence. The irregular 10:4 mode shape appears intermittently with long periods of quiescence between, behavior that was also generally typical of this mode shape for the other ramp angles (i.e., the 20:3, and 30:6 shapes). As the ramp angle increases, however, the prevalence of these quiescent periods decreases, suggesting that the increased panel curvature promotes the excitation of this specific shape. For the higher ramp angles, we also see generally similar trends in that the lowest frequency modes are excited throughout the majority of the test time, while the higher frequency modes are subject to more impulsive excitation.

**Fig. 23:**
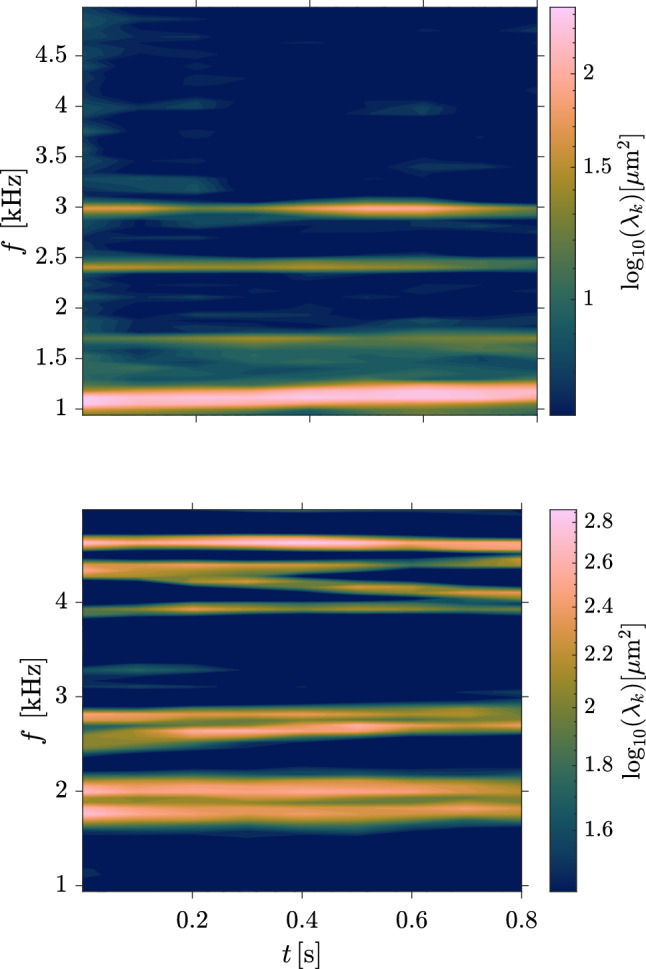
Transient panel modal frequencies using short-time SPOD algorithm on the out-of-plane displacement measurements for (top) $\theta = 10^\circ$, $\Delta p = 0$ kPa and (bottom) $\theta = 30^\circ$, $\Delta p = 0$ kPa. $\lambda _k$ represents the associated eigenvalue of the displacement signal, and thus the power present in each mode shape

To gain a better understanding of the temporal evolution of the frequencies of the various excited modes, we divide the out-of-plane panel motion data into 0.1 s segments with 50% overlap and compute the transient SPOD spectra; examples for both the $\theta = 10^\circ$ and $\theta = 30^\circ$ ramps at $\Delta p = 0$ kPa are shown in Fig. 23. For the $10^\circ$ ramp, the lower-frequency modes (i.e., the 10:1 and 10:2) tend to gradually grow in vibrational frequency throughout the recorded test time, though this effect is most prominent for the 10:1 mode. We observe a similar behavior for the lower modes of the $30^\circ$ ramp, primarily with the 30:1, 30:3, and 30:4. This increase can be attributed to stiffening resulting from the growing thermal-stress-induced deformation. Such increases in vibrational frequencies were generally observed for these lower-frequency modes over all ramp angles and $\Delta p$ values. The higher-frequency modes, in contrast, tend to exhibit a downward shift in frequency throughout the test time. We first see this to a minor extent with the 10:4 mode shape and more noticeably with the 30:6 and 30:8 modes. We also note an interesting branching behavior between the 30:6 and 30:7 modes, in which they initially overlap at the same vibrational frequency, before the 30:6 mode experiences a significant drop in $f$. This branching behavior was consistently observed for these two modes on the $\theta = 30^\circ$ ramp, though the time at which the divergence in frequency occurred was delayed for the higher $\Delta p$ experiments, suggesting that the static deflection shape influences this behavior. These downward shifts in frequency for certain mode shapes can be attributed to the thermal softening of the material (and thus a reduction in the modulus of elasticity Budiansky and Mayers 1956; Wang et al. 2024), and we infer that this effect tends to dominate the higher-frequency modes. There were cases though, such as the 30:4 and 30:7 modes, in which the frequency remained essentially constant over the recorded time. The relative dominance of thermal softening (which tend to decrease mode frequencies) and stress-induced panel curvature (which increases the frequencies LaFontaine et al. 2016; Pinho et al. 2023) thus appear to depend on the particular mode shape in a non-trivial manner.

**Fig. 24:**
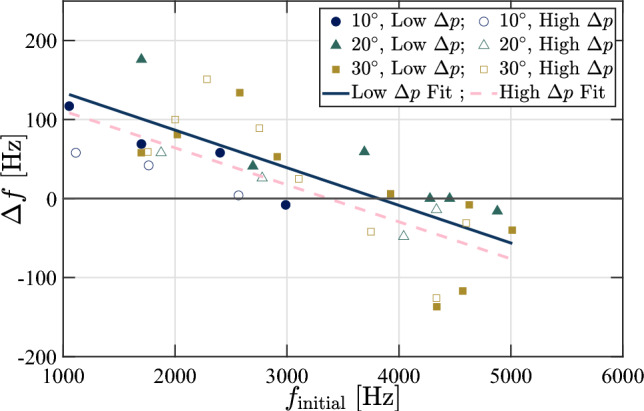
Shift in initial vibrational frequencies for the various ramp angles and extremes of the $\Delta p$ cases as a function of the initial vibrational frequency of each mode. The closed markers correspond to the lowest $\Delta p$ test cases while the open markers correspond to the highest $\Delta p$ test case for each ramp angle

To provide an overall comparison of the evolving modal characteristics between the different ramp angles and pressure differentials, we compute similar short-time SPOD spectra for each run condition and extract the frequency of each mode throughout the test time. In Fig. 24, we present the total change in frequency, $\Delta f$, as a function of the initial vibration frequency, $f_{\textrm{initial}}$, over the one-second recording time; data are presented only from the highest and lowest $\Delta p$ values for each ramp angle, as these bound the observed trends. Linear fits to each of the groups (high and low $\Delta p$) are also included for reference. The first trend we generally observe is that the lower-frequency modes ($f_{\textrm{initial}} \lesssim 2.4$ kHz) are more likely to increase in vibrational frequency throughout the test, whereas the opposite is true for the higher-frequency modes. This confirms the specific observations of Fig. 23 and reinforces the picture that the lower-frequency modes are more affected by the static deflection whereas the higher-frequency modes are more sensitive to thermal softening effects. The higher ramp angles do, on average, show larger magnitudes of $\Delta f$ (up to$\sim$10% for the lower-frequency modes), though only slightly. Finally, we see that the lower $\Delta p$ panels exhibit generally larger positive changes in frequency; we attribute this to the greater changes in static deflection, $\Delta \omega '$, experienced by these panels, as seen in Fig. 15.

**Fig. 25:**
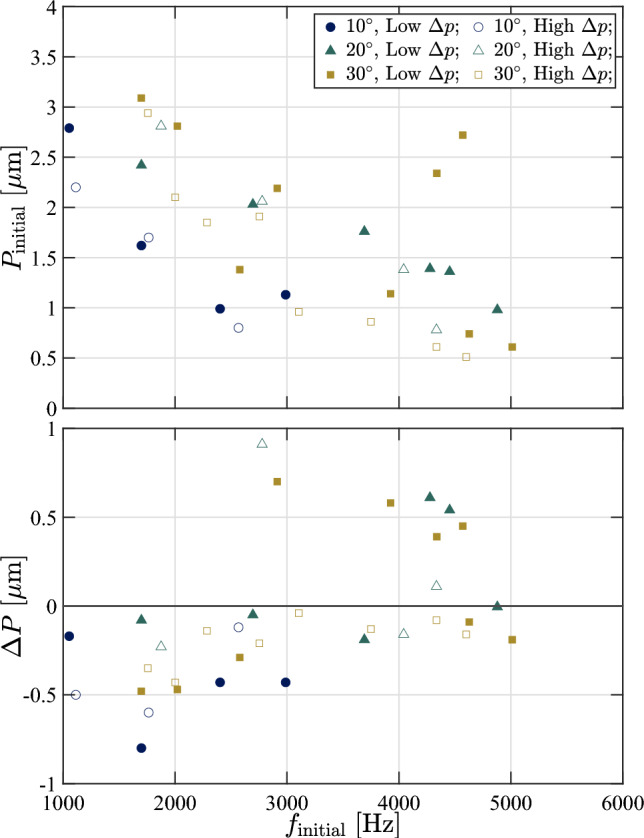
(Top) Initial modal power, $P$, and (bottom) shift in the modal power for the various ramp angles and extremes of the $\Delta p$ cases as functions of the initial vibrational frequency of each mode. The closed markers correspond to the lowest $\Delta p$ test cases while the open markers correspond to the highest $\Delta p$ test case for each ramp angle

We now examine the modal powers and their evolution for the various test conditions, as presented in Fig. 25. Here, we have computed the integrated spectral power of each mode (which we henceforth refer to as just the power) in the short-time SPOD directly by summing the eigenvalues within the full-width half-maximum of each peak in the spectrum. In the upper part of Fig. 25, we present the initial mode power, $\textit{P}_{\textrm{initial}} = \textrm{max}\left( \sqrt{\lambda _k}\cdot \Psi _k\right)$, for each ramp angle and the highest and lowest $\Delta p$ test cases as a function of the initial vibrational frequency. These powers represent the 2$\sigma$ mean of the vibratory amplitudes of the marker grid (Whalen et al. 2020). We note that as the vibrational frequency increases, the initial vibrational spectra RMS power generally decreases; this is generally consistent with the strengths of the peaks noted in the mean SPOD spectra in Figs. 16 to 18. Some prominent exceptions to this trend are seen for the highest ramp angle, however, in which the fluid forcing is the most unsteady and SWBLI strength is the highest. Regarding the effects of the pressure differential, a higher $\Delta p$ generally leads to a slight reduction in $P_{\textrm{initial}}$ (consistent with the mean SPOD spectra in Fig. 19), potentially due to the increased rigidity of the panel (LaFontaine et al. 2016; Freydin and Dowell 2021). In the bottom graph of Fig. 25, we show the change in the modal power, $\Delta P$, over the recording time as a function of the initial vibrational frequency. The behavior of this quantity generally mirrors that of $\Delta f$: those mode shapes that experienced an increase in frequency during the recorded time showing a decrease in power, and vice versa.

**Fig. 26:**
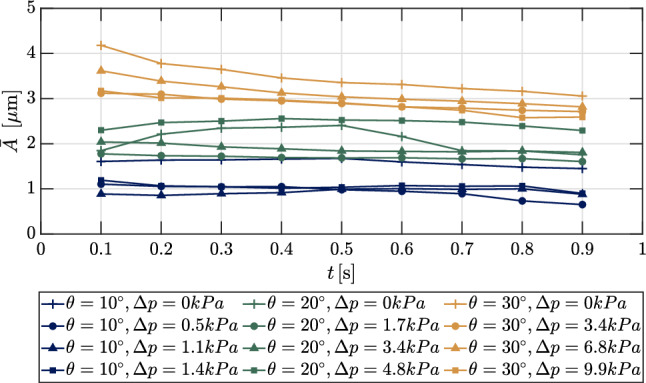
Evolution of the average frequency-integrated vibratory amplitude, $\bar{A}$, derived from the SPOD eigenvalues for all experiments

Finally, we inspect the evolution of the total vibratory power of the panel throughout each test. To do this, we sum the short-time SPOD eigenvalues between 500 Hz and 8000 Hz, subtract out the noise floor, and normalize by the number of marker points. Finally, we take the square root of this quantity, which now represents the effective average amplitude, $\bar{A}$, across the panel. These amplitudes are shown in Fig. 26. As might be expected, $\bar{A}$ grows monotonically with ramp angle, suggesting that the overall increasing mechanical loading for the stronger SWBLI tends to dominate the bulk panel dynamics over any particular targeting of individual mode shapes through impingement. The $\theta =30^\circ$ cases also show the largest decreases in $\bar{A}$ over the recorded time, indicating that deflection-induced stiffening is more important than thermal softening for the overall panel motion for this ramp angle. The other ramp angles show more modest decreases (and in some cases essentially none at all). The zero pressure differential cases for the $\theta =10^\circ$ and $30^\circ$ ramps tend to exhibit the highest average amplitudes, but other trends with $\Delta p$ aren’t clear. Overall, we found panel vibrations that are somewhat reduced compared to findings at Mach 6 by Whalen et al. (2020) and Schöneich et al. (2023), suggesting the larger static deformations of the present case introduce increased rigidity in the panel, restricting vibratory motion.

## Conclusions

In this work, the response of a clamped thin panel to a Mach-10, ramp-induced SWBLI has been characterized for a variety of ramp angles (10$^\circ$, 20$^\circ$, and 30$^\circ$) and panel pressure differentials. To resolve the unsteady, full-field panel dynamics, a new high-fidelity marker-tracking algorithm was introduced; this method was shown to outperform previous techniques, producing highly accurate centroid measurements and exhibiting robustness under high-noise conditions. Measurements of the underlying SWBLI flowfield using self-aligned focusing schlieren and fast-response pressure transducers showed that the incoming boundary layer was late transitional, and that the SWBLI was attached (or minimally separated) for 10$^\circ$ and separated for the larger ramp angles.

When combined with a Spectral Proper Orthogonal Decomposition analysis, the high-accuracy photogrammetry technique allowed both the frequencies and out-of-plane displacement distributions of the dominant panel modes to be uncovered under the various conditions. Although in some cases the regular (m,n) mode shapes from classical plate theory were present, these tended to be heavily distorted (presumably by the substantial streamwise pressure and thermal gradients across the panel) and also lay at somewhat higher frequencies than determined through pre-tunnel modal testing. We also observed mode shapes that differed quite significantly from those expected by classical theory; simulations revealed that these were introduced by the large static deformations present. Additional shifts in frequency were observed during the recorded test time, indicating that the already complex fluid-structural interaction induced by the SWBLI becomes more involved when considering the heating-induced transient shifts in the modal vibrational frequencies. Regardless of the origins and mechanisms causing the frequency shifting of the panel modes, this noticeable change has meaningful implications for both potential flow feedback as well as long-term panel fatigue considerations. We found that increased static deformations, likely caused by compressive thermal and normal strains, are the driving mechanism behind the upward shifts in the natural frequencies and seem to dominate the lower frequency vibrational modes. Further, probable thermal softening due to increased heating can explain the downward shift in some natural frequencies of the higher-frequency modes. Overall, the effect of nonzero pressure differential was to increase the stiffness of the panel, leading to an increase in modal frequencies and a decrease in power, particularly for higher-frequency modes.

Although the influence of the intense Mach-10 heating environment was inferred from the available measurements here, for future studies it would be recommended that direct measurements of the temporally and spatially resolved panel temperature be made, ideally through infrared thermography.

## Figures and Tables

**Fig. 27:**
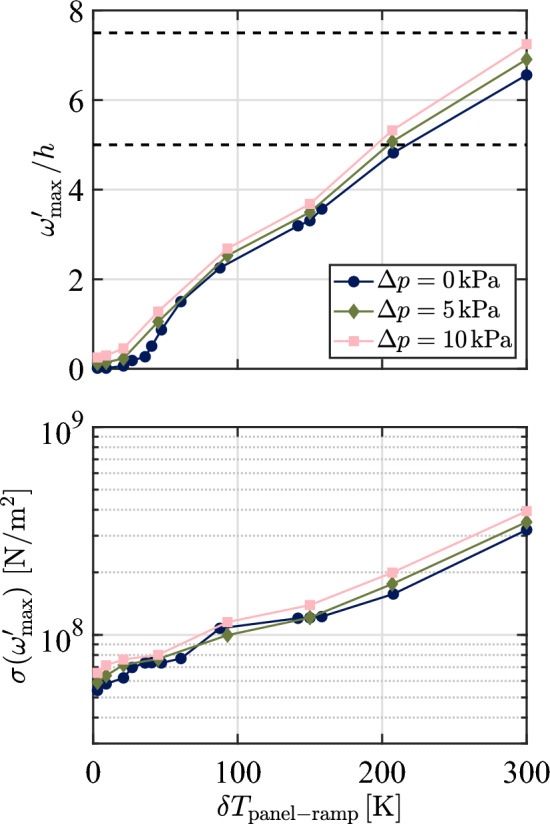
Development of (top) the maximum static deformation, $\omega '$, normalized by the panel thickness, h, and (bottom) the Von Mises stresses, $\sigma$, taken at the point of maximum static deformation, $\omega '$, at discrete $\delta T_\mathrm {panel-ramp}$ values for each condition. The dashed horizontal lines in the top graph encompass the range of $\omega _\textrm{max}'/h$ values seen in experiments

**Fig. 28:**
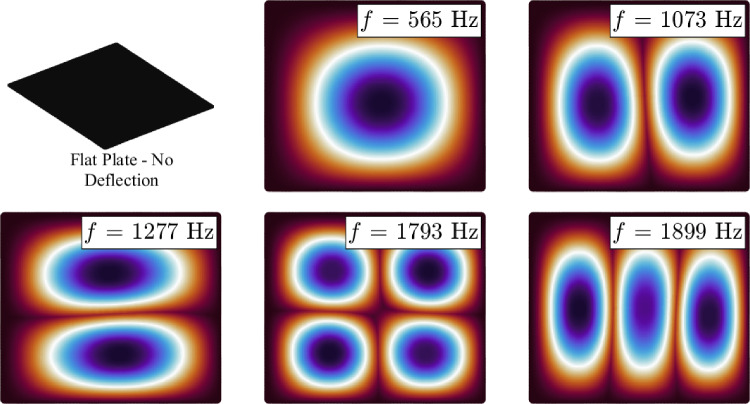
First five vibrational modes of non-deflected flat plate from SolidWorks^®^ simulation

**Fig. 29:**
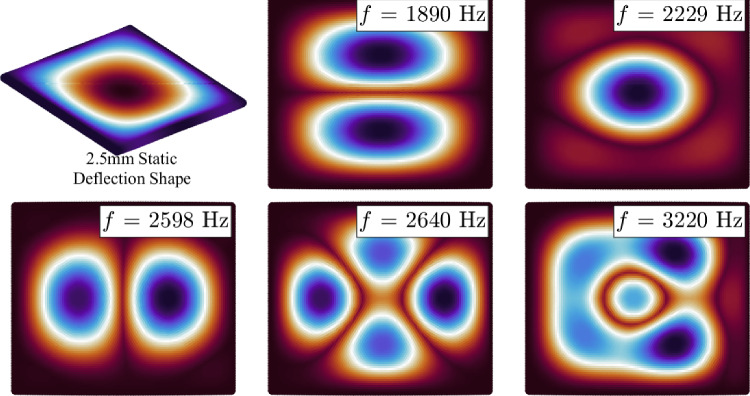
First five vibrational modes of 2.5 mm static deflection flat plate from SolidWorks^®^ simulation

**Fig. 30:**
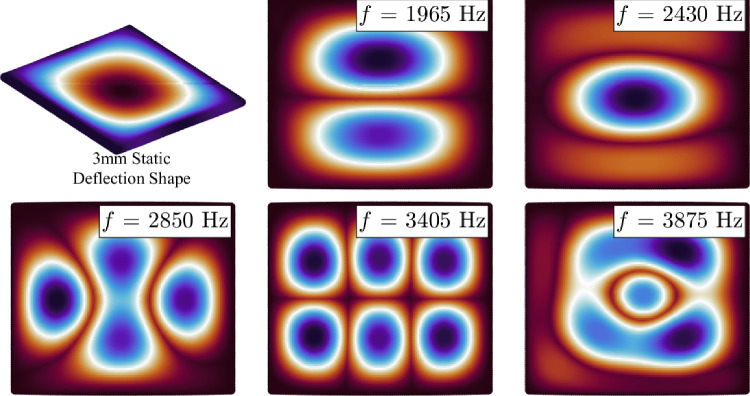
First five vibrational modes of 3 mm static deflection flat plate from SolidWorks^®^ simulation

**Fig. 31:**
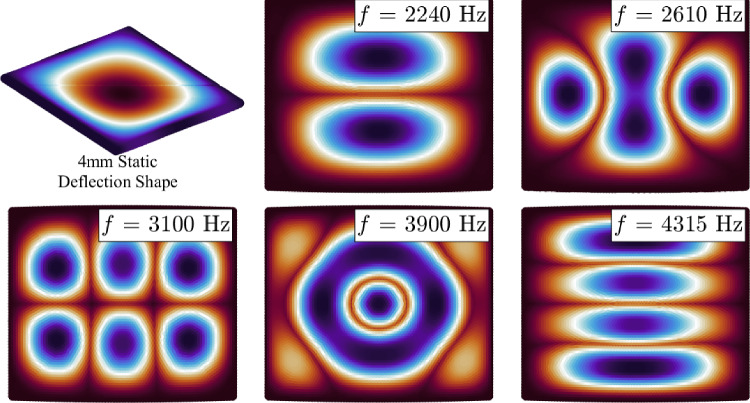
First five vibrational modes of 4 mm static deflection flat plate from SolidWorks^®^ simulation

## Data Availability

The data that support the findings of this study are available from the authors upon reasonable request.

## Appendix A

To assess the degree to which the thermal loading impacts the overall deflection, and thus internal stresses, of the compliant panel, SolidWorks’^®^ nonlinear static solver was utilized. The solver computes the steady-state equilibrium response of a structure to a prescribed load(s) and iteratively enforces force balancing using a Newton–Raphson scheme. To allow for large deflections, geometric nonlinearity is included via large-displacement kinematics, also allowing for stress redistribution to be captured. There simulations were used to examine the static deflection shape and internal stresses of the compliant panel deformed by both a surface temperature differential between the thin panel and the rigid housing, which we denote $\delta T_\mathrm {panel-ramp} (= T_\textrm{panel}-T_\textrm{ramp})$, and a pressure differential, $\Delta p$, across the panel. We chose $\delta T_\mathrm {panel-ramp}$ values ranging from 0 to 300 K and $\Delta p$ values of 0, 5, and 10 kPa. All surface temperatures and pressure differentials were applied uniformly across the ramp-panel configuration; although the real aero-thermal loading will be nonuniform, however, the results of these simulations should encompass the trends seen in experiments. A fixed boundary condition was applied to the panel sides, constraining all translational and rotational degrees of freedom, to simulate a rigid attachment to the surrounding structure. Material properties were given by the 15–5 PH/H1025 (AMS 5659) stainless steel ramp from the experiments. The panel began in a non-deformed, zero-stress state.

Beginning with the maximum normalized static deflection, shown in the top graph of Fig. 27, this increases monotonically with $\delta T_\mathrm {panel-ramp}$, as expected. The relationship appears to be fairly linear, though we note small initial growth in $\omega _\textrm{max}'$ for $\delta T_\mathrm {panel-ramp} \lesssim 40 \hspace{.05cm} \textrm{K}$. For $\delta T_\mathrm {panel-ramp}$ between 200 K and 300 K, we recover deflection values consistent with those seen in the present experiments. The presence of a pressure differential across the panel only serves to introduce a small initial deflection that remains relatively constant as the temperature differential increases. The bottom graph of Fig. 27 shows the Von Mises stress, $\sigma$, taken at the point of maximum static deflection for each case. We see the difference in $\sigma (\omega _\textrm{max}')$ between each $\Delta p$ case is minimal, indicating the majority of the panel stiffening effects and temporal changes observed in experiments can be attributed to the thermally induced stresses caused by the intense aero-thermal environment.

## Appendix B

SolidWorks^®^ frequency simulations were performed of both a flat plate with no deflection and curved panels with various maximum deflections, in all cases with fully clamped boundary conditions. In each instance, the first five mode shapes were extracted. For the flat plate, shown in Fig. 28, the mode shapes are as expected, corresponding to the (1,1), (2,1), (1,2), (2,2), and (3,1) modes from classical clamped-plate theory. For the curved panel, three maximum deflections of 2.5, 3.0, and 4.0 mm were simulated. To model these deflected cases, we projected the flat panel onto a spherical cap with varying radii to produce the three desired maximum deflections. A fixed boundary condition was applied to the panel boundaries, constraining all translational and rotational degrees of freedom of the selected surfaces to simulate a rigid attachment to the surrounding structure. For the 2.5 mm case shown in Fig. 29, we first note the disappearance of the (1,1) mode and a large rise in the vibrational frequencies for the mode shapes shared with the flat plate. This is likely due to the increased strains within the panel caused by the curvature. We also begin to see mode shapes that deviate noticeably from the classical (m,n) distributions: only the first and third modes for this case produce mode shapes with the standard horizontal and vertical nodal lines, with the other mode shapes giving unexpected spatial distributions due to the panel curvature. These irregular mode shapes continue to feature heavily as the panel curvature is increased (Figs. 30 and 31), though the largest-deflection case is somewhat distinct (with no evidence of the second mode shape present for the other two seen here).
